# Stone Age *Yersinia pestis* genomes shed light on the early evolution, diversity, and ecology of plague

**DOI:** 10.1073/pnas.2116722119

**Published:** 2022-04-12

**Authors:** Aida Andrades Valtueña, Gunnar U. Neumann, Maria A. Spyrou, Lyazzat Musralina, Franziska Aron, Arman Beisenov, Andrey B. Belinskiy, Kirsten I. Bos, Alexandra Buzhilova, Matthias Conrad, Leyla B. Djansugurova, Miroslav Dobeš, Michal Ernée, Javier Fernández-Eraso, Bruno Frohlich, Mirosław Furmanek, Agata Hałuszko, Svend Hansen, Éadaoin Harney, Alina N. Hiss, Alexander Hübner, Felix M. Key, Elmira Khussainova, Egor Kitov, Alexandra O. Kitova, Corina Knipper, Denise Kühnert, Carles Lalueza-Fox, Judith Littleton, Ken Massy, Alissa Mittnik, José Antonio Mujika-Alustiza, Iñigo Olalde, Luka Papac, Sandra Penske, Jaroslav Peška, Ron Pinhasi, David Reich, Sabine Reinhold, Raphaela Stahl, Harald Stäuble, Rezeda I. Tukhbatova, Sergey Vasilyev, Elizaveta Veselovskaya, Christina Warinner, Philipp W. Stockhammer, Wolfgang Haak, Johannes Krause, Alexander Herbig

**Affiliations:** ^a^Department of Archaeogenetics, Max Planck Institute for Evolutionary Anthropology, 04103 Leipzig, Germany;; ^b^Department of Archaeogenetics, Max Planck Institute for the Science of Human History, 07745 Jena, Germany;; ^c^Institute for Archaeological Sciences, Eberhard Karls University of Tübingen, 72074 Tübingen, Germany;; ^d^Biology and Biotechnology Faculty, Al-Farabi Kazakh National University, 050040 Almaty, Kazakhstan;; ^e^Institute of Genetics and Physiology, Al-Farabi Kazakh National University, Almaty, 050060 Kazakhstan;; ^f^Faculty of Mathematics and Computer Science, Friedrich-Schiller University, 07743 Jena, Germany;; ^g^Begazy-Tasmola Research Center of History and Archeology, 050008 Almaty, Kazakhstan;; ^h^Nasledie Cultural Heritage Unit, 355006 Stavropol, Russian Federation;; ^i^Research Institute and Museum of Anthropology, Lomonosov Moscow State University, 125009 Moscow, Russian Federation;; ^j^Department of Heritage Management, Archaeological Heritage Office Saxony, 01108 Dresden, Germany;; ^k^Department of Prehistoric Archaeology, Institute of Archaeology, Czech Academy of Sciences, 11801 Prague, Czech Republic;; ^l^Department of Geography, Prehistory, and Archaeology, University of the Basque Country, Vitoria-Gasteiz, 01006 Spain;; ^m^Department of Anthropology, National Museum of Natural History, Smithsonian Institution, Washington, DC 20560;; ^n^Institute of Archaeology, University of Wrocław, 50139 Wrocław, Poland;; ^o^Archeolodzy.org Foundation, 50316 Wrocław, Poland;; ^p^Eurasia-Department, German Archaeological Institute, 14195 Berlin, Germany;; ^q^Department of Organismic and Evolutionary Biology, Harvard University, Cambridge, MA 02138;; ^r^Department of Genetics, Harvard Medical School, Boston, MA 02115;; ^s^Faculty of Biological Sciences, Friedrich-Schiller University, 07743 Jena, Germany;; ^t^Evolutionary Pathogenomics, Max Planck Institute for Infection Biology, 10117 Berlin, Germany;; ^u^Institute of Ethnology and Anthropology, Russian Academy of Science, 119991 Moscow, Russian Federation;; ^v^Research Laboratory of Paleoanthropological Study, Institute of Archaeology named after A.Kh Margulan, Almaty, 50010 Kazakhstan;; ^w^History Department, Al-Farabi Kazakh National University, 050040 Almaty, Kazakhstan;; ^x^Centre for Egyptological Studies of the Russian Academy of Sciences, Russian Academy of Sciences, 119991 Moscow, Russian Federation;; ^y^Curt Engelhorn Center Archaeometry, 68159 Mannheim, Germany;; ^z^Transmission, Infection, Diversification & Evolution Group, Max Planck Institute for the Science of Human History, 07745 Jena, Germany;; ^aa^Institute of Evolutionary Biology, Consejo Superior de Investigaciones Cientificas-Universitat Pompeu Fabra, 08003 Barcelona, Spain;; ^bb^Department of Anthropology, University of Auckland, 01010 Auckland, New Zealand;; ^cc^Institute for Pre- and Protohistoric Archaeology and Archaeology of the Roman Provinces, Ludwig Maximilian University Munich, 80539 Munich, Germany;; ^dd^Department of Human Evolutionary Biology, Harvard University, Cambridge, MA 02138;; ^ee^BIOMICs Research Group, University of the Basque Country Universidad del Pais Vasco/Euskal Herriko Unibertsitatea, 01006 Vitoria-Gasteiz, Spain;; ^ff^Archaeological Centre, 779 00 Olomouc, Czech Republic;; ^gg^Department of Evolutionary Anthropology, University of Vienna, 1030 Vienna, Austria;; ^hh^Institute of Fundamental Medicine and Biology, Kazan Federal University, Kazan, 420008 Russian Federation;; ^ii^Laboratory for Structural Analysis of Biomacromolecules, Federal Research Center “Kazan Scientific Center of the Russian Academy of Sciences”, 420111 Kazan, Russian Federation;; ^jj^Department of Anthropology, Harvard University, Cambridge, MA 02138

**Keywords:** ancient DNA, plague, *Yersinia pestis*

## Abstract

The bacterial pathogen *Yersinia pestis* gave rise to devastating outbreaks throughout human history, and ancient DNA evidence has shown it afflicted human populations as far back as the Neolithic. *Y. pestis* genomes recovered from the Eurasian Late Neolithic/Early Bronze Age (LNBA) period have uncovered key evolutionary steps that led to its emergence from a *Yersinia pseudotuberculosis*-like progenitor; however, the number of reconstructed LNBA genomes are too few to explore its diversity during this critical period of development. Here, we present 17 *Y. pestis* genomes dating to 5,000 to 2,500 y BP from a wide geographic expanse across Eurasia. This increased dataset enabled us to explore correlations between temporal, geographical, and genetic distance. Our results suggest a nonflea-adapted and potentially extinct single lineage that persisted over millennia without significant parallel diversification, accompanied by rapid dispersal across continents throughout this period, a trend not observed in other pathogens for which ancient genomes are available. A stepwise pattern of gene loss provides further clues on its early evolution and potential adaptation. We also discover the presence of the flea-adapted form of *Y. pestis* in Bronze Age Iberia, previously only identified in in the Caucasus and the Volga regions, suggesting a much wider geographic spread of this form of *Y. pestis*. Together, these data reveal the dynamic nature of plague’s formative years in terms of its early evolution and ecology.

## Significance

The bacterium *Yersinia pestis* has caused numerous historically documented outbreaks of plague and research using ancient DNA could demonstrate that it already affected human populations during the Neolithic. However, the pathogen’s genetic diversity, geographic spread, and transmission dynamics during this early period of *Y. pestis* evolution are largely unexplored. Here, we describe a set of ancient plague genomes up to 5,000 y old from across Eurasia. Our data demonstrate that two genetically distinct forms of *Y. pestis* evolved in parallel and were both distributed across vast geographic distances, potentially occupying different ecological niches. Interpreted within the archeological context, our results suggest that the spread of plague during this period was linked to increased human mobility and intensification of animal husbandry.

The earliest known cases of human infection with the plague pathogen, *Yersinia pestis*, date to around 5,000 y ago (1–4). Analyses of ancient *Y. pestis* genomes from this period suggest that the time window between 6,000 and 4,000 y ago was critical and formative for the evolution and ecology of *Y. pestis* as we know it today. Four ancient *Y. pestis* lineages have been identified so far, which can be genomically distinguished based on their adaptations to the flea, the main vector of modern plague. Today, fleas are known to play a central role in the transmission of plague within rodent populations, which can act as reservoirs from where spillovers to human populations typically occur (5, 6). The transmission of *Y. pestis* by the flea is either facilitated by a blockage of the foregut (proventriculus), where the bacterium produces a biofilm (7), or in a biofilm-independent manner, also known as early-phase transmission (8, 9). The oldest lineages of *Y. pestis* (2, 4) (hereafter referred to as preLNBA−), and the Late Neolithic and Early Bronze Age (LNBA−) lineage (1, 3) present a genetic background that has been interpreted as being incompatible with flea transmission via the blockage of the foregut (indicated in the naming by the minus sign). While a recently identified ancient lineage also dating to the Bronze Age presents all the genetic adaptations for this highly efficient form of flea transmission (10) (LNBA+; the plus sign indicates the adaptation to the flea vector). Intriguingly, both variants coexisted for millenia and they might have occupied different niches. However, it remains unclear how the different forms of *Y. pestis* infected humans during prehistory and how the resulting diseases manifested in the human population. Whether plague ecology and transmission as we know it today can serve as a model to understand its manifestation in the past remains also unknown.

Elucidating the ecology and transmission will be crucial for understanding how the LNBA+/− lineages of plague, which were widespread across Eurasia for thousands of years (1, 3, 10, 11), have impacted human societies, and how changes in human subsistence and economy have shaped the early evolution of this pathogen. It is currently unknown whether and which types of animal populations served as potential reservoirs of the disease and their identification will be essential for characterizing past *Y. pestis* transmission dynamics. The absence of an adaptation to the flea vector in some plague lineages suggests that the transmission dynamics were complex. Today, the flea-mediated model is not the only documented form of plague transmission: pneumonic plague can be acquired via respiratory droplets from close human-to-human contact. However, only a few reported outbreaks have been attributed to this transmission mode and usually in contexts of poor ventilation and direct contact with infected individuals (12–17). Additionally, plague has been documented in humans who handled or ingested parts of infected animals (18–22).

Changes in human behavior may also have contributed to a higher risk of plague infection. During the LNBA period, archeological evidence attests to technological advances, such as the spread of oxen-drawn carts and wagons (23) and horse domestication (24), which enabled increased human mobility and exploitation of new habitats, such as the Eurasian steppe belt. This ultimately led to the establishment of long-distance networks, in which raw materials such as copper were circulated (25, 26). However, periods of unrest and war could also have played a role in the extended human mobility during the LNBA period. While earlier studies hypothesized that increased mobility was the cause for an early spread of *Y. pestis* across Eurasia (1, 3), it could also have been its effect. It is also during this period that animal husbandry and mobile pastoralism intensified in the steppe (27), thus facilitating the overlap of ecological niches for zoonoses to occur. The aforementioned changes could have played a role in the likelihood of transmission to humans and the long-distance spread of plague during its early evolution.

Here we expand the number of *Y. pestis* genomes from the LNBA period to offer a higher genomic resolution for important stages in the evolution of the bacterium, as well as its diversity and geographical distribution in the past. By linking the genomic evidence with the available archeological context, we discuss potential transmission mechanisms of plague during its early evolution.

## Results

### Screening and Genome Reconstruction.

**Fig. 1.**
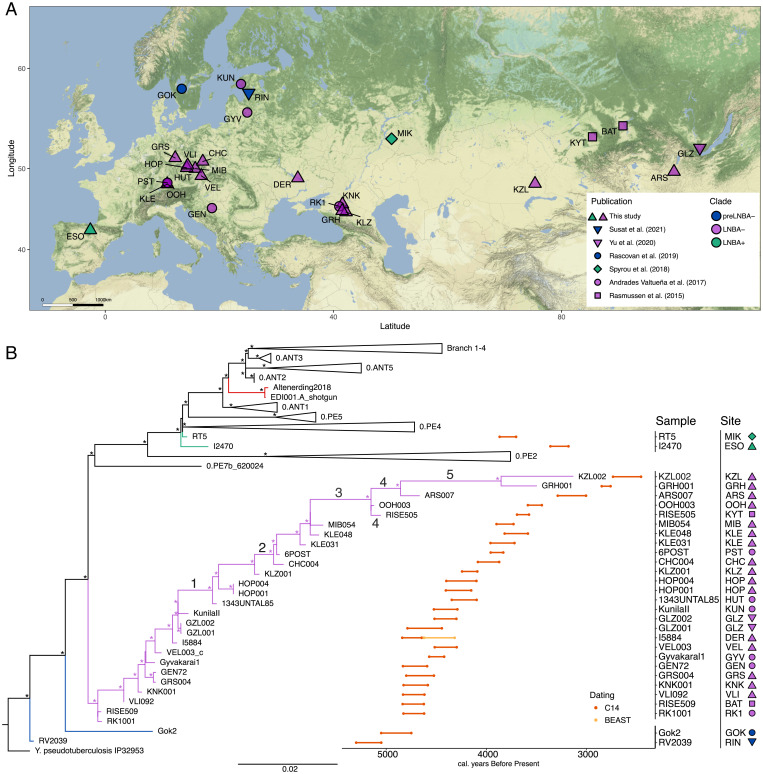
Sampling locations, phylogeny and radiocarbon date ranges of newly reported and relevant published *Y. pestis* genomes. (*A*) Archeological sites where *Y. pestis* genomes have been recovered dating to the LNBA period. A list of the site names and abbreviations can be found in *SI Appendix*, Table S1. (*B*) ML tree computed from all variable positions (SNPs) in *Y. pestis* (*n* = 7,506); the uncollapsed tree can be seen in *SI Appendix*, Fig. S1. Unique positions to the outgroup (*Y. pseudotuberculosis*) were excluded from the SNP alignment to improve visibility. The scale represents the expected number of substitutions per site. Numbers on the tree indicate the deletions detected in the genomes displayed in *SI Appendix*, Fig. S5. Colored are ancient branches that appear to be extinct today: blue indicates the preLNBA− lineages, purple the LNBA− lineage, green the LNBA+ flea-adapted genomes from the Bronze Age, and red the genomes from the first plague pandemic. Nodes marked with asterisks have a bootstrap support of at least 90. The plotted date interval on the right corresponds to radiocarbon 2σ date ranges (C14; dark orange) or 95% HPD dates intervals (light orange) inferred by BEAST of the genomes from the LNBA period aligned to the corresponding tips in the ML tree. Symbols and colors correspond to those in *A*. Plots were produced with ggplot (39), ggmap (40), ggalt (41), and ggpubr (42) packages with R v3.6 (43); the phylogenetic tree was plotted with FigTree v1.4.4 (https://github.com/rambaut/figtree/releases/tag/v1.4.4) and Inkscape (44) was used for the final figure.

We screened a total of 252 samples from 15 archeological sites that span from Western Europe to the eastern Eurasian steppe, dating from the Late Neolithic until the Iron Age (∼5,000 to 2,000 y ago) (Fig. 1*A* and *SI Appendix*, *Archeological Information* and Table S1) using the HOPS pipeline (28) for the presence of *Y. pestis* DNA. Candidates for capture enrichment for *Y. pestis* DNA were identified as those where: 1) reads aligned to *Y. pestis* or the *Y. pseudotuberculosis* complex, 2) we observed a decrease in the number of reads aligned when the number of mismatches increases (decreasing tendency observed in the edit distance distribution), 3) an ancient DNA damage pattern was detected, and 4) manual inspection of the alignments in MEGAN (29) revealed an even distribution of the reads across the reference. Targeted DNA enrichment permitted the reconstruction of 17 ancient *Y. pestis* genomes with coverages ranging from 7.5 to 30.6x, 19.1 to 66.3x, 7.9 to 38.2x, and 28.8 to 154.9x for the chromosome, pCD1, pMT1, and pPCP1 plasmids, respectively (see *SI Appendix*, Table S2 for the chromosome, and Dataset S1 for the plasmids). A sample originally published as RISE139 in Rasmussen et al. (1), CHC004 in this study, was also included; while the sequencing of 487 million reads in the original publication yielded 0.14x, 0.28x, 0.24x, and 0.76x coverages for the chromosome, pCD1, pMT1, and pPCP1 plasmids, sequencing of 33,542,357 reads after capture performed here increased the coverage to 8x, 21.4x, 10.1x, and 59x, respectively. This highlights the economical use of capture techniques to recover *Y. pestis* genomes even when low levels of the pathogen DNA are present in shotgun sequencing data.

### Sixteen Newly Reconstructed Genomes Phylogenetically Placed within the LNBA Lineage.

To assess the phylogenetic relationship of the newly recovered genomes to other *Y. pestis* strains, we computed a maximum-likelihood (ML) phylogeny, including modern representatives as well as previous ancient genomes from the Neolithic to the Bronze Age period (1–4, 10, 11), as well as from the first (30, 31) and second (32–35) plague pandemics (Dataset S2).

Sixteen of the 17 newly reconstructed genomes fall into the previously reported LNBA− lineage (Fig. 1*B* and *SI Appendix*, Fig. S1). Genomes of this lineage have been reported from present-day Russia (Caucasus, Lake Baikal, and Altai region), Germany, Poland, Croatia, Estonia, and Lithuania (1, 3, 11). We now report the presence of this pathogen in the Czech Republic, Ukraine, Eastern Kazakhstan, and Mongolia, thus extending the known geographical expanse of *Y. pestis* in the past. As previously shown (1, 3, 11), the genomes within the LNBA− lineage branch in a clocklike fashion in order of their mean calibrated radiocarbon date, with the exception of I5884 (*Materials and Methods* and Fig. 1*B*). Given that I5884 is phylogenetically more derived on the LNBA− lineage than Gyvakarai1 and VEL003, which date to 4,571 to 4,422 and 4,516 to 4,297 calibrated years before present (cal. BP), respectively, we would expect the C14 dating range of I5884 to either overlap with those genomes or to be younger. Instead, we observe an older, nonoverlapping date (4,840 to 4,646 cal. BP) for I5884. This unexpected age could be explained by a reservoir effect, which results in shifts of the C14 dates. Such effects can occur through the consumption of marine or freshwater resources, whereby, via different factors, such as deep geological filtering and consumption habits of the involved fish, carbon in these foodstuffs is derived from geologically older sources of carbonates rather than atmospheric carbon.

In order to address this, we took advantage of the high correlation (*R*^2^ = 0.971) between the age and the root-to-tip distance of the samples present in the LNBA− lineage (*SI Appendix*, Fig. S2). This method has been previously used to estimate radiocarbon date offsets caused by a reservoir effect in GLZ001 and GLZ002, where the BEAST dating agrees with the isotopic correction (11). We estimated the molecular date of I5884 to 4,579 to 4,371 y BP (95% highest posterior density [HPD]) (*SI Appendix*, Fig. 3) using BEAST v2.6.6 (36), which is in line with the expected calendar date based on the phylogenetic position (Fig. 1 *B* and *C*). This offset corresponds well to the ∼250 y reservoir effect reported from one of the Dereivka I Neolithic graves (37, 38). The KZL002 genome (Kazakhstan) recovered here dates to 2,736 to 2,457 cal. BP, placing it within the Iron Age. To our knowledge this is the youngest genome recovered from the LNBA− lineage and shows that this lineage survived for at least 2,500 y. Despite its long-term persistence, there is a lack of known modern representatives, which leads to the assumption that the lineage went extinct sometime after the Iron Age.

### First Evidence of Prehistoric Plague on the Iberian Peninsula.

Intriguingly, we were also able to reconstruct a novel genome from an individual found in the dolmen “El Sotillo” in Álava (Spain, I2470). This represents the first evidence of prehistoric plague in the Iberian Peninsula dating to 3,361 to 3,181 cal. BP. Despite a radiocarbon date that places it as contemporaneous with some of the youngest genomes in the LNBA− lineage (e.g., ARS007), it occupies a different position in the phylogeny. Though approximately 500 y younger, the I2470 genome branches off basal to the previously reported RT5 genome from the Samara region in Russia (10), which remains the oldest genome identified to have the full suite of genetic features required for flea-based transmission and having been capable of causing bubonic plague. The I2470 genome thus represents another lineage of flea-adapted plague in Europe, highlighting the diversity of strains present in Eurasia shortly after the possible emergence of *Y. pestis*. The fact that we observe these two lineages with bubonic potential (LNBA+) at opposite ends of Europe, raises questions on how widespread the flea-adapted forms were during this period across Eurasia and how quickly the dispersal of these variants occurred across this vast territory.

### Temporal Coexistence of *Y. pestis* Lineages with Different Transmission Potential.

**Fig. 2.**
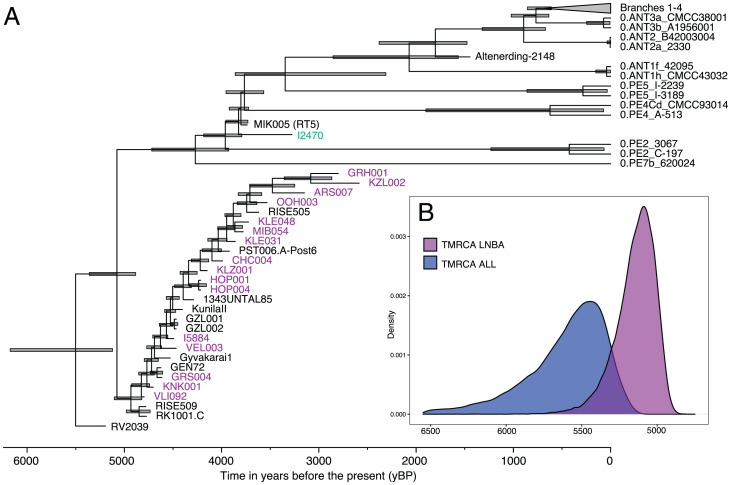
*Y. pestis* molecular dating using BEAST. (*A*) Maximum-clade credibility tree summarizing the results of divergence dating analysis between all extant *Y. pestis* lineages and the LNBA− lineage based on the coalescent skyline tree prior in BEAST v2.6.6. The maximum-clade credibility tree was produced using TreeAnnotator and visualized using FigTree v1.4.4 (https://github.com/rambaut/figtree/releases/tag/v1.4.4). Newly generated genomes are shown in purple (LNBA−) and green (I2470). (*B*) Posterior estimates of the time to the most recent common ancestor (TMRCA) for the divergence of all known *Y. pestis* as well as the divergence of the LNBA− clade are shown for the coalescent skyline tree prior. Density plots were produced using the ggplot2 package (39) in R v3.6 (43).

To investigate the divergence timing between the LNBA− lineage and all extant *Y. pestis*, we performed a molecular dating analysis with the Bayesian statistical framework BEAST v2.6.6 (36). For this, we used a selection of modern and historical *Y. pestis* genomes representative of all described phylogenetic clades [as in Bos et al. (45)] and all prehistoric genomes with >3-fold coverage (see Methods). A regression analysis comparing the root-to-tip distance with specimen age revealed a correlation coefficient (*r* = 0.44) acceptable for molecular dating analysis (*Materials and Methods*). Molecular dating was performed using the coalescent skyline tree prior, and revealed overlapping date estimates for the divergence between LNBA− lineage and the rest of the *Y. pestis* tree (95% HPD spanning between 6,174 and 5,122 y BP) (Fig. 2*A* and *SI Appendix*, Table S3). These estimates are consistent with those published previously (1–3), and suggest an initial diversification of *Y. pestis* during the Neolithic and Bronze Age periods. In addition, the split time of the LNBA− lineage appears contemporaneous with that of the most deeply divergent extant *Y. pestis* lineages (see 0.PE7, 0.PE2, 0.PE4, and 0.PE5, in Fig. 2*A* and *SI Appendix*, Table S3), suggesting a parallel diversification of multiple clades that followed different histories, and likely had different transmission and disease potentials. Finally, the split times of the recently published and newly sequenced ancient flea-adapted strains (RT5 and I2470) span the period between 3,957 and 3,723 y BP (*SI Appendix*, Table S3), which is also in line with previous estimates (10) and confirms their temporal coexistence with the LNBA− lineage.

### Genomic Content of *Y. pestis* During the LNBA Period.

**Fig. 3.**
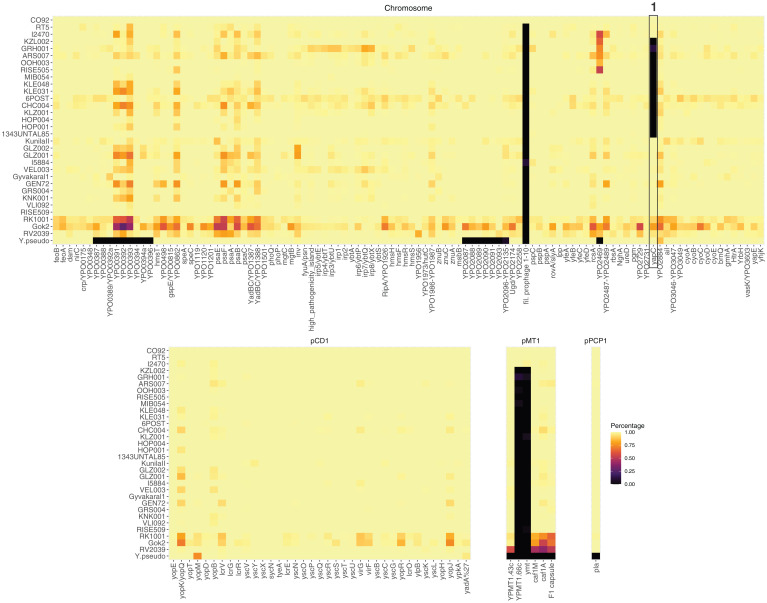
Status of known *Y. pestis* virulence factors in newly reported genomes. Heatmaps displaying the presence or absence of 159 known virulence factors (Dataset S3) in *Y. pestis* genes of the chromosome (*n* = 115), and the pCD1 (*n* = 37), pMT1 (*n* = 6), and pPCP1 (*n* = 1) plasmids. Yellow represents 100% of the gene covered at least 1X while black represents 0% of the gene covered. Genomes are ordered based on their phylogenetic placement with the outgroup *Y. pseudo* (*Y. pseudotuberculosis* IP32593) at the bottom. The numbered box highlights the yapC gene, whose loss is part of deletion event 1 (*SI Appendix*, Fig. S5). The heatmaps were produced using the ggplot2 (39) and ggpubr (42) packages in R v3.6 (43).

Since virulence potential is fundamental for mode and tempo of geographic diffusion, we evaluated the status (presence/absence) of known *Y. pestis* virulence factors present in the reference genome (*Y. pestis* CO92) for the strains reported here (Fig. 3). In the case of the Iberian genome I2470, we observe the complete set of known virulence factors in both the chromosome and *Y. pestis* specific plasmids, confirming that this genome is, like RT5, adapted to the flea vector. One exception is the absence of the chromosome-encoded filamentous prophage that has only been consistently incorporated in the genomes of 1.ORI strains (46). After visual inspection of the mapped reads, we also confirm the ancestral, less-efficient *pla* variant in I2470 (*SI Appendix*, Fig. S4), which was previously reported in RT5 (10) and all the other LNBA− genomes (1, 3, 11). In contrast, all new genomes within the LNBA− lineage also lack the *ymt* gene, important for the flea infection (47), as well as *YPMT1.66c*, a virulence factor involved in resistance to mammalian innate immunity (48). The lack of those genes and the presence of active *ureD* and biofilm regulators (*SI Appendix*, Fig. S4), which have been previously reported in the LNBA− lineage (1, 3), suggest this lineage is a nonflea-adapted form of plague. We also observe the absence of the *yapC* gene in the 1343UnTal85 genome and all subsequent genomes.

Substantial genetic loss has been previously identified in some strains of the LNBA− lineage (1, 3). We systematically evaluated the presence of deletions across the *Y. pestis* CO92 reference genome for all LNBA− strains. We detected multiple deletions bigger than 500 bp across genomes of the LNBA- lineage, which can be grouped into five loss events in chronological order containing mostly membrane and flagellar proteins (Fig. 1*B*, *SI Appendix*, Fig. S5 and Table S4, and Dataset S4): the oldest event (event 1) occurred in the ancestor of 1343UnTal85 and involved the loss of a 35 kb region, which contains, among others, the *yapC* gene (Fig. 3); this was followed by event 2, the loss of a 1.5 kb region in the ancestor of CHC004 (RISE139); a third region (event 3) of 2 kb was lost in the ancestor of OOH003 and RISE505; a larger deletion comprising 37 kb (event 4) was detected in the genomes RISE505, ARS007, GRH001, and KZL002; and finally, event 5 occurred in the ancestor of GRH001 and KZL002, comprising various regions of the CO92 genome that totaled ∼83 kb. Event 4 may provide further insights into the relationship of OOH003, RISE505, ARS007, GRH001, and KZL002 genomes. The phylogenetic algorithm used here groups OOH003 and RISE505 in a clade that is ancestral to ARS007, GRH001, and KZL002 (Fig. 1*B*). Based on this topology, the deletion event 4 would have occurred independently in the lineage, leading to RISE505 and in the lineage that gave rise to ARS007, GRH001, and KZL002. Alternatively, the deletion represents supporting evidence for RISE505, ARS007, GRH001, and KZL002 forming a clade, which had lost the 37 kb region after its split from the ancestor of OOH003. The latter requires a single event to describe the presence of the deletion and thus is a more parsimonious explanation.

In terms of groups of genes contained in these deletions, we find that event 4 contains almost exclusively flagellin genes and event 5 contains genes related to the type VI secretion system (T6SS), particularly parts of the T6SS-G secretion system, the loss of which has been associated with attenuation (49). However, this was inferred based on a mutant defective for the *vasK* gene (50). The *vasK* gene is present in the LNBA− strains (Fig. 3), thus making inferences of the potential attenuation of the LNBA− strains difficult. The presence of flagellin genes in the deletions could also speak for adaptative evasion of the immune system by LNBA− strains. However, functional studies testing the specific genes found absent in that lineage would be required to infer their virulence potential.

Regarding the mechanism that might have caused these deletions, some of them can be explained by the presence of insertion sequence elements surrounding them (event 1 and 4) (*SI Appendix*, Table S4), which has been linked to deletions and rearrangements in *Y. pestis* (51). However, for some others, such as event 2, event 3, and parts of event 5, we have no direct indication of a specific mechanism, although we cannot exclude the involvement of insertion sequence elements since the genome arrangement may have differed to that of the reference. We caution that we can only detect the presence/absence of known genetic variation present in comparison to *Y. pestis* CO92, which was used as reference. Therefore, novel genetic elements exclusively present in the genomes presented here, as well as their genomic arrangement, could not be evaluated.

To evaluate the potential effect of single nucleotide polymorphisms (SNP) specific to the LNBA− branch, we performed a SNP effect analysis with SNPEff v3.1 (52). We detect a total number of 892 SNPs found only in the LNBA− branch (Dataset S5). Of those, 444 SNPs are either intergenic (*n* = 161, Dataset S6) or synonymous (*n* = 283, Dataset S7) and probably represent neutral changes. In contrast, we observe the presence of 429 nonsynonymous SNPs (Dataset S8) that could affect protein function due to amino acid changes, the effect of which, however, is hard to predict with genetic information alone. We detect 19 substitutions that likely lead to pseudogenization: one lost stop codon, three lost start codons, and the gain of 15 stop codons (Dataset S9). As with the deletions, we observed an accumulation of pseudogenes over time (*SI Appendix*, Fig. S6, Dataset S10) that appears to happen at a higher rate in the LNBA− branch than in the other basal branches. Interestingly, two of the affected genes (*flgB* and *fliZ*) are involved in flagella synthesis or are part of the flagellar system that is inactivated in all extant *Y. pestis*, probably as an adaptation to evade host immune response (53). While pseudogenization of *fliZ* is only detected in MIB054, the gain of an early stop codon in *flgB* is present in genome 1343UNTAL85 and all younger genomes of the LNBA- lineage, until the gene is completely lost as part of the larger genomic deletion first observed in RISE505 (event 4) (Fig. 1*B*, *SI Appendix*, Fig. S5, Dataset S4, and Dataset S9).

### LNBA Genomes Derive from a Single Lineage.

The current diversity and genomic make-up of LNBA− genomes show different characteristics compared to the flea-adapted lineages, which are responsible for more recent plague epidemics. To test whether the genomes in the monophyletic LNBA− branch evolved from a single population that provided a perpetual source deme of the pathogen without parallel diversification, we explored the potential correlation between genetic versus geographical distance and genetic versus temporal distance. The rationale is based on the following three assumptions: 1) we expect to see a correlation between geography and genetic affinity when genomes from the same location are genetically closer to each other, indicating the presence of multiple populations restricted to certain geographical areas; 2) for a single population evolving through time, we also expect a correlation between genetic affinity and temporal distance; 3) if no correlation is observed between geography and genetic distance or between time and genetic distance, this suggests a globally distributed diversity of the bacterium from which we randomly sampled any given clade at any given time. We compared our results to three additional ancient bacterial datasets (*SI Appendix*, Table S5): 1) *Y. pestis* genomes dating to the second pandemic that emerged from the Black Death clone (32, 34, 35) and form part of a European lineage, 2) *Salmonella enterica* (54–56), and 3) *Mycobacterium leprae* (57–59).

**Fig. 4.**
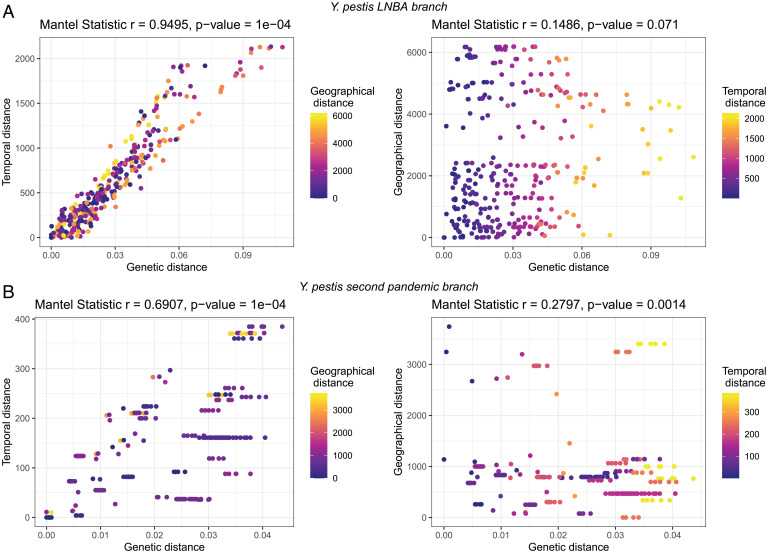
Genetic, temporal, and geographical distance correlations in ancient *Y. pestis*. Correlations between temporal (years) and genetic distance with colors indicating the geographical distance (kilometers) (*Left*), and geographical and genetic distance with colors indicating temporal distance (*Right*) for *Y. pestis* datasets: (*A*) LNBA− genomes (*n* = 26), (*B*) Second pandemic genomes (*n* = 27). Each dot represents the pairwise distance between two samples. Mantel statistics were calculated using the vegan (60) package. Distances matrices were plotted using ggplot2 (39) and ggpubr (42) packages in R v3.6 (43).

Comparison of the results from all four cases under study reveals a strong positive correlation between genetic distance and time (Mantel statistic *r* = 0.9495) in the LNBA− genomes, indicating that these arose from a single lineage. We also observed no contribution to the genetic distance explained by geography (Fig. 4*A*), which speaks toward a high mobility for this lineage. In the case of the second pandemic *Y. pestis* genomes, we expected to see a weak correlation between genetic and temporal distance due to parallel lineages evolving through time and no correlation between geography and genetic distance, since we know that a single clone was responsible for the Black Death that spread across a large geographic area of Europe with local diversification (35). We found this assumption confirmed by a weak but significant correlation between genetic distance and time (Fig. 4*B*), but we also observed a weak but significant correlation between genetic and geographic distance, which could be related to potentially distinct reservoirs that formed during the second plague pandemic (35, 60). Similarly, we also observed a weak correlation between genetic and temporal distance in *S. enterica* (*SI Appendix*, Fig. S7*C*), since most of the ancient strains derive from a few lineages within the diversity of this pathogen (54). In contrast, we observed nonsignificant *P* values in correlations for *M. leprae* (*SI Appendix*, Fig. S7*D*). This is probably due to the fact that contemporaneous reconstructed genomes are distributed across the phylogeny of the species, independent of their location or age (57, 59), and thus reveals what to expect in a scenario in which a globally distributed pathogen underwent parallel evolution.

## Discussion

Ancient *Y. pestis* genomes recovered from humans who lived between 5,000 and 2,500 y BP have revealed key evolutionary features in the early evolution of this pathogen (1–4, 10, 11). Currently, the earliest evidence of plague in humans dates to as early as 5,300 y BP, a time when three different lineages have been detected in Eurasia: a genome from Latvia representing the most basal *Y. pestis* identified to this date (4); a strain found in Sweden (2) that is chronologically close to the basal strains in the LNBA− lineage located in the North Caucasus and Altai mountains (1, 3); and the LNBA− lineage itself (Fig. 1*B*). These early lineages lacked genetic adaptations shown to be essential for the efficient transmission of this bacterium via the flea vector, namely the *ymt* gene (47), the silencing of both biofilm regulators (61), and the pseudogenization of *ureD* (62) (Fig. 3 and *SI Appendix*, Fig. S4). This led to the initial hypothesis that flea-based transmission arose from genetic changes acquired during the Iron Age (1). This assumption was challenged by the recovery of a fully flea-adapted strain (RT5) from Russia dating to 3,800 y BP, temporally overlapping and thus coexisting with the LNBA− lineage (10). Here, we provide further evidence for flea-adapted (LNBA+) strains during the Bronze Age through the identification of this form of the bacterium in an Iberian individual (I2470), which postdates RT5 by approximately 500 y. While the full geographic expanse of the flea-adapted form during the Bronze Age is still unknown, these two genomes, located ∼5,000 km apart, suggest that this form was already widespread across Europe. Moreover, our results show that a diversity of *Y. pestis* lineages was present across Eurasia shortly after the emergence of all known *Y. pestis* strains, which we date to as early as ∼6,200 y BP. However, given the scarcity of available data from flea-adapted genotypes, details on the emergence and dispersals of LNBA+ variants remain to be explored.

Early plague diversity is not only characterized by genomic variation but also by potential differences in ecology and transmission. For the LNBA+ ancient lineages, represented by RT5 and I2470, we can assume a transmission cycle similar to that observed in modern contexts (lineages 0.PE7, 0.PE2, and 0.PE4), where fleas serve as vectors of the disease and maintain the transmission of the bacterium in host rodent populations (63). The formulation of hypotheses about means of transmission for the more basal *Y. pestis* lineages is more challenging. One of the limitations for this inference is the current lack of close modern relatives with similar genetic characteristics, which strongly suggests that those lineages have since become extinct. Furthermore, all ancient *Y. pestis* genomes have been recovered from humans, thus limiting our interpretations in terms of the host range of past strains. Understanding which nonhuman host and vector species were involved in LNBA− *Y. pestis* ecology, if any, becomes fundamental for the inference of the transmission of these strains. *Y. pestis* can infect a wide range of mammals, with rodents being the main reservoir of the disease. Other species, such as carnivores, domesticates, or birds could potentially spread the disease into other regions (64, 65). However, we regard this as rather unlikely, since for modern plague these species have been reported only to be involved in short distance dispersal and usually represent dead-ends for the transmission of the bacterium. Regarding paleo-epidemiological patterns in the human population, we observed no indication of major human outbreaks nor changes in mortuary practices. In contrast, all individuals diagnosed as plague-positive in this study were buried in accordance with local burial customs, indicating that the cause of death was not perceived as unusual. This could indicate that humans were not the only sustaining hosts of the disease, unless the LNBA− strains caused a less severe form of plague.

Even if humans were not sustaining the disease, the fact that we observed the presence of the LNBA− lineage across Eurasia raises the question whether humans were involved in the dispersal of plague during this period, and whether human mobility provided opportunities for the bacterium to exploit new ecological niches. The time period around 5,000 y ago is characterized by an intensification of human mobility across Eurasia, attested by the expansion of pastoralist groups both eastward and westward from the Eurasian steppes (66, 67). The emergence of new subsistence forms, such as mobile dairy pastoralism (68, 69), and the extended use of new forms of transport, such as oxen-drawn carts and wagons (23) and the subsequent domestication of horses (24), aided in the increased mobility of humans (25, 26). Given that the Eurasian steppe served as a corridor for the connection between geographically distant human populations, especially in combination with intensified and expanding pastoralism during this period, suggests increased contact or habitat overlap between wild animals, such as rodents, and humans and their livestock. Livestock is occasionally infected by plague (18, 21, 22, 70, 71) and in rare events can act as intermediate hosts in human cases of plague (71). The intensification of pastoralism in the grasslands of the steppe (68) could have served not only as a zone of interaction between humans, livestock, and sylvatic hosts, but also have increased the chances of transmission. In combination with increased human mobility this might have facilitated the connection of ecosystems and habitats that otherwise would not have come into contact, therefore creating opportunities for the dispersal of diseases into and across new territories and hosts.

In addition, we have shown that the LNBA− genomes form a single lineage that experienced very little parallel diversification through time, potentially indicating a single or well-connected reservoir of the disease that would allow for a high mobility of strains with frequent replacement events, and from which zoonotic events could have occurred regularly. The wide geographic spread of the LNBA− lineage and the fact that it also reached regions beyond the steppe (e.g., mixed temperate forest zones in Central Europe, the Altai and Lake Baikal regions), speaks for intensified mobility among wild animals and humans with their domesticates. However, whether a scenario with a transmission chain involving wild and domestic animals in the past appears conceivable remains an open question, particularly as the LNBA− lineage was likely not able to be efficiently transmitted by fleas.

From a genomic perspective, we observed an increase in the pseudogenization and genetic loss during the evolution of the LNBA− lineage starting around 4,200 y ago. This could be an indication of strong selection pressure in the bacterial population (72) or a sign of adaptation to new hosts (73, 74). The blocked-flea mechanism employed by *Y. pestis* requires genetic adaptations that allow it to colonize and block the flea foregut, resulting in increased bite frequency and enhanced transmission of the bacterium. LNBA− strains lack the required adaptations for this type of transmission. However, these strains could still have been transmitted by fleas, albeit inefficiently, since the recently described “early-phase transmission” also permits flea-mediated infection in the absence of blockage (8, 9, 75, 76). Furthermore, a recent study has shown that the *ymt* gene is not essential for survival in the flea gut, depending on the origin of the blood meal (77). The authors suggest that *ymt* missing strains had a more restricted host range, which is in line with a low level of parallel diversification as indicated by the strong correlation between genetic and temporal distance in the LNBA− strains. Spillovers of the LNBA− strains into other hosts would have resulted in evolutionary dead-ends given their potentially restricted host range. On the other hand, the *Y. pestis* strains carrying the *ymt* gene (LNBA+) would have been able to establish new reservoirs in a wider range of hosts, thus being more competitive than the LNBA− strains. This could be a possible explanation for the extinction of the LNBA− lineage.

Another potential route for transmission is the oral−fecal route, which is the main transmission path for the *Y. pestis* ancestor, *Y. pseudotuberculosis*. However, the LNBA− *Y. pestis* strains would likely have had a higher capacity to cause a systemic disease than its ancestor, since the *pla* gene, involved in dissemination of the bacterium in the mammalian host (78), had already been acquired. Additionally, consumption of infected animals is also an oral route of *Y. pestis* transmission for which various reports exist: for example, from camels (18, 21, 22), goats (21), and marmots (19). Finally, it has been suggested previously that the initial form of plague was pneumonic in its nature (79). This is the rarest form of plague today (12–17), but cases of pneumonic plague infection via the inhalation of blood droplets during the process of skinning plague-infected animal carcasses have been documented (80). While all of these transmission scenarios are possible, more research is needed to address this question.

Overall, we observed the long-term coexistence in western Eurasia of two forms of *Y. pestis* (a fully flea-adapted and a nonflea-adapted form), which likely lasted for at least 2,500 y. Whether these forms competed in the same ecological niche, coexisted among the same hosts, or occupied entirely different niches requires further examination. In addition, questions remain about the dispersal history and the full geographic expanse of the flea-adapted form. For the nonadapted form, further ancient genomes from the LNBA period, particularly those recovered from animal remains, combined with functional studies that evaluate their genetic characteristics, would be fruitful avenues of future research to better characterize the transmission mechanisms of early forms of plague.

## Materials and Methods

For a detailed description of the archeological sites, experimental methods, and data analysis, refer to *SI Appendix*.

### Data Generation, Screening, and Enrichment of *Y. pestis* DNA.

We screened a total of 252 individuals from 15 sites from Eurasia dating between ∼5,000 and 2,000 y BP. Teeth were sampled and DNA was extracted as described in refs. 81–84. Extracts were further processed into double-indexed double-stranded Illumina sequencing libraries (85) with partial removal of the deaminated sites with USER enzyme (86) using the protocol described in Aron et al. (87) and referred as half-UDG treated samples from now on. The laboratory process for samples from the Dereivka I site and dolmen “El Sotillo” have been previously described in refs. 81 and 88, respectively. For the samples from Velešovice and Grushevskoe, single-stranded libraries were prepared using the automated protocol described in Gansauge et al. (89).

All libraries were shotgun-sequenced to 5 million reads on an Illumina HiSeq 4000 (single-end kit −1 × 76 + 8+8 cycles) at the Max Planck Institute for the Science of Human History,Jena or a NextSeq500 (paired-end kit −2 × 76 + 7+7 cycles) at the Harvard Medical School, Boston and screened for the presence of *Y. pestis* DNA using the HOPS pipeline (28). Positive samples were then enriched for *Y. pestis* DNA following the in-solution capture described in Andrades Valtueña et al. (3). Additional single-stranded libraries were also prepared for OOH003, KNK001, KLZ001, and ARS007 following the aforementioned protocol and enriched for *Y. pestis* DNA with in-solution capture as explained above. Sequencing of the enriched libraries was done on either a HiSeq4000 or NextSeq500.

### Data Processing, Variant Calling, and Phylogeny.

Raw sequencing reads were processed with nf-core/eager (90) (v2.2.2), with the exception of I5884 and I2470 samples that required a preprocessing step to remove the 7-bp internal barcodes (*SI Appendix*). In short, adapters were trimmed with AdapterRemoval v2.3.1 (91). For half-UDG–treated samples, 1 bp was clipped from both ends of the read with FASTX-trimmer v.0.0.14 (http://hannonlab.cshl.edu/fastx_toolkit/) to remove potential bias due to deaminated cytosines. The resulting reads were aligned to the *Y. pestis* CO92 chromosome (NC_003143.1) and plasmids (pCD1: NC_003131.1, pMT1:NC_003134.1 and pPCP1: NC_003132.1) with bwa v0.7.17 aln (92). Duplicated reads were removed with Picard Tools v1.140 MarkDuplicates (93); bam files from the same individual were merged and used to calculate mappings statistics and perform variant calling with GATK UnifiedGenotyper v.3.5 (94).

The final SNP alignment was produced with MultiVCFAnalyzer v0.85.2 (95) (https://github.com/alexherbig/MultiVCFAnalyzer), including the newly generated samples as well as other ancient and modern genomes (Dataset S2). SNP calls identified as false positives in the prehistoric *Y. pestis* genomes were excluded (Datasets S11–S13). An additional genotyping for the single-stranded genomes was performed with GenoSL (https://github.com/aidaanva/GenoSL) (*SI Appendix*). The resulting snpAlignment was used to compute a ML tree with RAxML-NG (v0.9.0, https://github.com/amkozlov/raxml-ng)

### Molecular Dating Analyses.

Given the incongruence between the phylogenetic positioning and radiocarbon date of I5884 (Fig. 1 *B* and *C*), we applied a molecular dating approach using the program BEAST v2.6.6 (36) with a dataset including all genomes from the LNBA− branch and the branch 0 strain 0.PE2 Pestoides F (used as outgroup) to reevaluate the specimen’s age (*SI Appendix*, *Bayesian Molecular Dating of I5884*).

To estimate the divergence time between the LNBA− clade and all other *Y. pestis* diversity, we used the Bayesian phylogenetic framework implemented in BEAST v2.6.6 (36). For this, we compiled a dataset including all described prehistoric strains with greater than threefold average coverage and a subset of genomes representing all *Y. pestis* clades described to date [genome selection as in Bos et al. (45)]. After confirming an acceptable correlation (*r* = 0.44) in root-to-tip regression analysis (*SI Appendix*, *Molecular Dating Analysis*), we performed a molecular dating analysis (see *SI Appendix*, *Molecular Dating Analysis* section for details on set-up) with two demographic models: constant coalescent and the coalescent skyline. Path sampling was used to evaluate which of the two models was more suitable for the data (*SI Appendix*). The coalescent skyline model was strongly favored and was used in the final dating. Two independent chains (300,000,000 states) were run for each of the demographic models. Results were viewed in Tracer v1.6 (http://tree.bio.ed.ac.uk/software/tracer/) to ensure run convergence (all posterior effective sample sizes > 200) and to ensure posterior estimate consistency. A maximum-clade credibility tree was created using TreeAnnotator with a 10% burn-in, which was visualized and edited in FigTree v1.4.4 (https://github.com/rambaut/figtree/releases/tag/v1.4.4).

### Virulence Analysis and Indel Analysis.

To assess the presence and absence of known virulence factors in *Y. pestis*, we compiled a bed file containing the coordinates for genes on the chromosome (*n* = 115), and the pCD1 (*n* = 37), pMT1 (*n* = 6), and pPCP1 (*n* = 1) plasmids of *Y. pestis* CO92 (Dataset S3). In order to account for regions that may have mapability issues (e.g., duplicated regions), we mapped the trimmed reads and the sslib reads as above with the exception that no mapping quality filter was applied (–bam_mapping_quality_threshold 0). The output bam files were then used to calculate the percent of the gene covered using bedtools v2.25.0 (96) and prepared the data for R using Generate_bed_files.sh. The resulting bed files were concatenated using the cat command and the final files can be found in https://github.com/aidaanva/LNBAplague/tree/main/Data/Virulence. The results were plotted in R (43) using the ggplot2 package (39).

Additionally, we used the resulting nonfiltered bam files to explore the presence of chromosomal deletions using *Y. pestis* CO92 as reference. We recovered noncovered regions from bam files as follows: bedtools genomecov was used to calculate the noncovered regions per sample; noncovered regions separated by less than 100 bp were then merged together and subsequently filtered to have a minimum size of 500 bp. We also calculated the percentage of coverage for each missing window to account for sparse data in low-coverage genomes. The resulting files per sample were then combined and analyzed with R. Additionally, we extracted the genes affected by any deletion. All of these steps were implemented in the script IndelCheck.sh. For the missing regions, we plotted deleted regions containing less than 15% of the region covered using the ggplot2 (39) and ggalt (41) packages.

### Phylogeography and Temporal Testing.

To test whether the genomes in the LNBA− lineage are indeed descendants of one another, we tested whether there is a correlation between either genetic and geographical distance or genetic and temporal distance. We performed this analysis in R by calculating the genetic distance as the pairwise distance using the dist.dna function of the ape package (97) and as input the filtered snpAlignment.fasta from MultiVCFAnalyzer to contain only the LNBA− genomes and their variable sites. The geographic coordinates were collected from each of the archeological sites used in this study (https://github.com/aidaanva/LNBAplague/blob/main/Data/2020-07-09_LNBA_leprosy_enterica_comp/LNBA_transect/Metadata_coordinates_dating_sex_updated_def.csv) and pairwise linear distances were calculated as the shortest distance between two geographical points using the distm function of the geosphere package (98). Finally, the median of each calibrated radiocarbon date (2-sigma, in years BP) was used to calculate the temporal pairwise distances using the outer function from base R. We performed mantel statistics to test whether there was a correlation between genetic versus geographic distance matrices or genetic versus temporal distance matrices. This was performed using the mantel function from the vegan (99) package in R. The correlations were plotted using ggplot2.

In order to provide comparative data for these correlations, we performed the same analysis using high-coverage genomes from the second plague pandemics, ancient leprosy (*M. leprae*) and ancient *Salmonella* data (*SI Appendix*, Table S5) (see https://github.com/aidaanva/LNBAplague/tree/main/Data/2020-07-09_LNBA_leprosy_enterica_comp subfolders for the data). The final figure was generated in R using the ggpubr package (42).

All the previously described R code can be found in the R notebook here: https://github.com/aidaanva/LNBAplague/blob/main/Stone_Age_Plague_v5.Rmd.

## Supplementary Material

Supplementary File

Supplementary File

Supplementary File

Supplementary File

Supplementary File

Supplementary File

Supplementary File

Supplementary File

Supplementary File

Supplementary File

Supplementary File

Supplementary File

Supplementary File

Supplementary File

## Data Availability

The data have been deposited in the European Nucleotide Archive, https://www.ebi.ac.uk/ena/browser/home (project no. PRJEB51099). All scripts and code mentioned can be found at https://github.com/aidaanva/LNBAplague (100). Previously published data were used for this work (*SI Appendix*, Tables S1 and S5).
