# Supplementary material for: Stone Age Yersinia pestis genomes shed light on the early evolution, diversity, and ecology of plague
Source: Proc Natl Acad Sci U S A. 2022 Apr 12;119(17):e2116722119. doi: 10.1073/pnas.2116722119 (PMC9169917; doi:10.1073/pnas.2116722119)
Supplement: Supplementary File [file pnas.2116722119.sapp.pdf]

Supplementary Appendix: Stone Age *Yersinia pestis* genomes shed light on the early evolution, diversity and ecology of plague

Supplementary Appendix: Stone Age Yersinia pestis genomes shed light on the early evolution, diversity and ecology of plague 1
Archeological Information 2
Arbulag soum (ARS, latitude (lat): 49.65 - longitude (lon): 99.72, Mongolia) 2
Chociwel (CHC, lat: 50.81 - lon: 17.09, Poland) 2
Grushevskoe (GRH, lat: 45.00 - lon: 41.54, Russia) 3
Großstorkwitz (GRS, lat: 51.18 - lon: 12.26, Germany) 3
Hostivice-Palouky (HOP, lat: 50.08 - lon: 14.28, Czech Republic) 4
El Sotillo (ESO, lat: 42.57 - lon: -2.62, Spain) 4
Dereivka I (DER, lat: 48.91 - lon: 33.76, Ukraine) 6
Kleinaitingen (KLE, lat: 48.22 - lon: 10.84, Germany) 6
Kaluzhnyy 1 (KLZ, lat: 44.84 - lon: 42.21, Russia) 7
Krasnogvardeyskoe (KNK, lat: 45.82 - lon: 41.58, Russia) 7
Kyzyl (KZL, lat: 48.27 - lon: 75.35, Kazakhstan) 8
Mikulovice (MIB, lat: 49.99 - lon: 15.77, Czech Republic) 8
Oberottmarshausen (OOH, lat: 48.23 - lon: 10.86 Germany) 9
Velešovice (VEL, lat: 49.17 - lon: 16.84, Czech Republic) 9
Vlineves (VLI, lat: 50.37 - lon: 14.44, Czech Republic) 9
Methods 11
Data generation 11
Pathogen Screening 12
Y. pestis enrichment 12
Data processing 13
Variant calling, SNP effect and phylogenetic analysis 13
Bayesian molecular dating of I5884 15
Molecular dating analysis 16
Virulence analysis and indel analysis 17
Phylogeography and temporal testing 17
Supplementary Figures 19
Supplementary Tables 27
Datasets 35
SI References 36

Archeological Information

Arbulag soum (ARS, latitude (lat): 49.65 - longitude (lon): 99.72, Mongolia)

Contact persons: Bruno Frohlich, Judith Liddleton

An 850 km2 area located in the Eastern steppe in the Arbulag soum (district) district found in the northern part of the Khovsgol aimag (province) in Mongolia has been surveyed for the presence of burial mounds by the Smithsonian Institution and the Mongolian Institute of Archaeology. Between 2006 and 2009, these institutions organized various excavation campaigns targeting the identified burial mounds (1–3). A total of 29 Bronze Age individuals were excavated dating to 3500- 2700 cal BC (1). Various animal bones were found in the burials, including horse, deer and bovids bones. Of those, 22 individuals were previously analyzed for their human DNA as well as for the presence of dairy proteins in their calculus (4). This revealed the presence of milk proteins from various ruminants (sheep, goat and bovidae) showing the long association of dairying practices in Mongolia, as well as, pointing to a pastoralist-based subsistence (4). From the previously 22 analyzed individuals, 11 individuals were included in the screening for the presence of *Yersinia pestis* DNA. The *Y. pestis* positive individual, ARS007 (archaeological ID 2007-9), is a 10-15 years old child whose biological sex has been determined anthropologically and genetically as male (3, 4).

Chociwel (CHC, lat: 50.81 - lon: 17.09, Poland)

Contact persons: Mirosław Furmanek, Agata Hałuszko

Chociwel site 1 is located NW of the modern village Chociwel, in the northern part of the Strzelin Hills (a microregion in the Sudetic Foreland, SW Poland) ca. 30 km south of Wrocław. The rescue excavations preceding the development of a sewage treatment plant were initiated in 1993 (5) and continued in 1994, 1995, 1998, 2011/2012 (P. Cholewa, A. Błażejewski, I. Lasak, K. Jarysz, P. Jarysz, R. Jarysz) (6–9). Funnel Beaker, Globular Amphora culture and early Medieval settlement remains (5, 7), as well as Early Bronze Age Únětice culture burial ground (6, 8) and an urnfield cemetery of the Lusatian culture (6) were discovered.

12 Early Bronze Age inhumations were found. All graves contained remains of single individuals. Most of them were oriented along the E-W axis; only one was aligned differently (N-S). Most of the individuals were buried in contracted position, on the right side, facing S. Burial good sets consisted of 1-5 ceramic vessels placed around the skull and chest or legs of the individual. The chronology of the discovered burials was based on typo-chronological data (6, 8) and narrowed down by radiocarbon dating (9, 10). This allowed to associate the burials with different phases of the Únětice culture: from the Old to Post-Classical phase. Analyses of stable isotopes suggest that the majority of these individuals had an omnivorous diet and represented a local population (11).

Samples from 6 individuals from the Únětice culture cemetery had been screened for aDNA during this project. This included the re-examination of 4 individuals already processed by the

RISE Project (10, 12). The presence of *Yersinia pestis* was found in one sample: CHC004 (RISE139), from feature 20 – a destroyed burial found during the 2011 excavation season. The grave contained the remains of an adult male aged 35-45 years (confirmed by genomic analysis), dated to 2137-1926 calBCE (3645±33 BP, Ua-44034) (10, 12) and equipped with a single undecorated pot (9).

Grushevskoe (GRH, lat: 45.00 - lon: 41.54, Russia)

Contact persons: Alexandra O. Kitova, Rezeda I. Tukhbatova, Egor Kitov
Grushevskoe fortified settlement, 391 burials, VII-VI centuries BC, Stavropol region, Russia

Ground burial with a total area of 14,357 sq. m. was excavated in 2018. The specified territory was explored down to the continental layers. A total of 391 burials and 38 objects were examined. A detailed description can be found in the archeological report (13).
About 2/3 of the burial complexes consisted of stone boxes and burials in pits. The rest of the burial complexes were discovered by the contrasting filling of the pits. The position of the skeletons is overwhelmingly crouched (in the fetal position or the sleeping position).
Burials 31, 60, 99, 100, 103, 159, 272, 292 were enclosed in stone rings (cromlechs).
Most of the burials contained inventory (grave goods). The bones stretched out on the back were buried without any inventory. The found grave goods are characteristic for the late stage of the Koban archeological culture.
The Koban culture (c. 1100 to 400 BC) is a late Bronze Age and Iron Age culture of the northern and central Caucasus. It is preceded by the Colchian culture of the western Caucasus and the Kharachoi culture further east (14, 15). The preliminary chronology of the investigated part of the burial ground is the 7th-6th centuries BC.
305 skeletons were previously sampled for an aDNA investigation, one of them (GRH001) was included in the present study for the screening of *Y. pestis* resulting in a positive detection of the pathogen DNA.
GRH001 was sampled from burial 13 - it was a pit of irregular shape with a chaotically scattered accumulation of stones of various sizes and irregular shapes. The skeleton was badly destroyed. Only a few fragments of leg bones, a jaw with teeth, lying in a cross at the eastern side, have survived. It was impossible to do sex and age determination. The tooth (GRH001) was radiocarbon-dated (14C Age [yr BP] - 2707, cal BC 896-815 cal BC 899-811).

Großstorkwitz (GRS, lat: 51.18 - lon: 12.26, Germany)

Contact persons: Matthias Conrad, Harald Stäuble

The site Großstorkwitz is located south of Leipzig within the Elbe-Saale-loess region. Within an area of more than one hectare, some 1500 features from Early Neolithic to the Middle Age were excavated by the Archaeological Heritage Office Saxony on account of the construction of a pipeline during 1996/1997. Alongside a large amount of settlement features 17 burials dating to the Final Neolithic Period (11 Corded Ware Complex, 1 Bell Beaker Complex, 5 unclassified) and four graves of the Early Bronze Age were found.

In this study, we screened one individual from the late Neolithic and three from the early Bronze Age for the presence of pathogen DNA. The individual GRS004 (archaeological ID PEG-07/499) who was tested positive for *Y. pestis* was a 35–45 years old person whose biological sex has been determined anthropologically and genetically as a male. The orientation of the crouched body was west-east, facing southwards. Only a flint blade was deposited as a grave good. A 14C analysis of the left femur of the individual dated the individual tp 4093±17 BP (Hd-21977) and is attributed to the early Corded Ware Complex resp. to the so-called Kalbsrieth group.

Hostivice-Palouky (HOP, lat: 50.08 - lon: 14.28, Czech Republic)

Contact persons: David Daněček, Jana Klementová, Miluše Dobisíková, Michal Ernée

A more detailed site description alongside the genome-wide data from the respective individuals is published in Papac et al. 2021 (16) and Daněček and Klementová 2008 (17). This site is located in the Prague-West district in central Bohemia and was part of a rescue excavation in 2007-2008 by J. Klementová and D. Daněček, covering an area of 10 ha. The occupation of the site started at least during the Linear and Stroked Pottery cultures and lasted throughout the Funnel Beaker, Řivnáč cultures, Hallstatt (Ha C-D1) and Roman Iron Age until the Early Middle Ages.
Four skeletons from graves associated with the Bell Beaker culture (features 688, 689, 690 and 691) were sampled for aDNA, two of which were positive for *Y. pestis*:
HOP001 (grave 17, feature 688; NM Prague Inv. No.: P7A 18906) was an adult male (35-50 years old), buried left-sided in a crouched position in north-south orientation, together with a cup and a bowl as grave goods. He was radiocarbon dated to 4405–4152 cal BP (2-sigma, MAMS-30798, 3837±24 BP).
HOP004 (grave 22, feature 691, NM Prague Inv. No.: P7A 18909) was a female of 40-60 years, buried in south-north orientation in a crouched position, atypically on the left side. She was radiocarbon dated to 4400-4100 cal BP (2 sigma, MAMS-38921, 3826±27 BP).

El Sotillo (ESO, lat: 42.57 - lon: -2.62, Spain)

Contact persons: Javier Fernández-Eraso, José Antonio Mujika-Alustiza

A detailed description of this site can be found in Lipson et al. 2017. It consists of a dolmen that was discovered in 1955 by Domingo Fernández Medrano, who excavated it in 1963 together with José Miguel Barandiaran and Juan María Apellániz. It forms part of the series of nine dolmens of the Rioja Alavesa located between the river Ebro and the Sierra Cantabria. On its southern slope, there are eight rocky shelters, some with an exclusively funerary nature (San Juan ante Portam Latiman), while the others alternated this use with that of pens or temporary shelter (Peña Larga, Los Husos I and II, San Cristóbal, Peña Parda and Balanciego I and II). These shelters have chronologies that range from the Late Neolithic, such as that of Peña Larga (6720 ± 40 BP, Beta.-242783, 5710-5610 cal BC), until the Final Bronze being contemporary with the individuals dated in El Sotillo. Those include Balanciego I (3110 ± 30 BP, Beta-339340, 1430-1370 / 1360-1310 cal BC), Los Husos I (2970 + 50 BP, Beta 136040, 1375-1020 cal BC) and San Cristóbal. Sup. Hole. Band 8 (3120 ± 40 BP, Beta-288940, 1460-1310 cal BC.). The study of the remains of fauna and pollens recovered in the shelters of the Sierra indicate the existence of a production economy

based on domestic animal species such as sheep, goats, bovids, as well as agriculture based on the cultivation of wheat and barley (18–20). Since the beginning of the Neolithic occupations, there has been a decrease in wooded areas with a decline in species such as yew and the advance of others such as hazelnut.

During the excavations of the El Sotillo dolmen, the remains of 13 individuals were exhumed that were radiocarbon (C-14) dated in the Beta Analytic laboratory in Miami (USA), which resulted in a range between 3096-813 cal BC (21). The complete results are shown in the following table:

Lp.	Laboratory code	Age (14C BP)	cal BC (95,4%)
1	Beta.- 299308	2740±30	971-813<br>971 (2,6%) 956<br>933 (92,8%) 813
2	Beta.- 299303	3120±30	1492-1291<br>1492 (1,5%) 1482<br>1450 (94,0%) 1291
3	Beta.- 299307	3160±30	1502-1323<br>1502 (92,3%) 1390<br>1336 (3,1%) 1323
4	Beta.- 299309	3320±30	1680-1509<br>1680 (4,9%) 1654<br>1642 (90,5%) 1509
5	Beta.- 299312	3360±30	1740-1539<br>1740 (10,7%) 1712<br>1696 (84,7%) 1539
6	Beta.- 299302	3360±30	1740-1539<br>1740 (10,7%) 1712<br>1696 (84,7%) 1539
7	Beta.- 299311	3380±30	1747-1544<br>1747 (90,3%) 1607<br>1581 (5,1%) 1544
8	Beta.-299101	3430±30	1875-1626<br>1875 (12,4%) 1843<br>1821 (4,6%) 1798<br>1778 (78,4%) 1626
9	Beta.- 299310	3550±30	2014-1771<br>2014 (2,0%) 1999<br>1976 (63,1%) 1868<br>1851 (30,3%) 1771
10	Beta.- 299300	4000±40	2630-2351<br>2630 (93,1%) 2451<br>2421 (0,9%) 2406<br>2377 (1,4%) 2351
11	Beta.- 299306	4040±30	2663-2469<br>2663 (1,5%) 2651<br>2632 (93,9%) 2469
12	Beta.- 299305	4350±30	3077-2899<br>3077 (2,7%) 3064<br>3027 (92,8%) 2899
13	Beta.- 299304	4390±30	3096-2913<br>3096 (95,4%) 2913

The set of dates indicates a usage of the tomb in three different stages that include the Chalcolithic, the Bronze Age and a last burial at the end of that period or the beginning of the Iron Age.
Two previous studies have analyzed the human DNA from 3 individuals from the Neolithic (22) and 6 from the Bronze Age (23). In this study, we screened 2 of the individuals included in the latter study.
I2470 is a genetically male individual (23), who is positive for *Y. pestis*, and has been dated to 3160 ± 30 BP (Beta 299307, 1502-1323 cal BC).

Dereivka I (DER, lat: 48.91 - lon: 33.76, Ukraine)

Contact persons: Ron Pinhasi, Sergey Vasilyev, Elizaveta Veselovskaya

A more detailed site description can be found in Mathiesson et al. 2018 (24) and Telegin 1991 (25). The site of Dereivka I is located close to the village with the same name in Ukraine, in the proximity of the Dnieper River. It is a Mariupol type cemetery that was excavated between 1961-1967 (25). A total of 173 burials were found, consisting of single and multiple graves. The majority of the individuals were found in a supine position. Previous ancient DNA studies have been conducted to study human DNA, in which 23 individuals were analyzed (24). From those, 20 individuals were included in the present study. We detected one positive individual for plague:
I5884 (Grave 68, feature 294) is an adult male individual, aged 45-55 years. It was found in a stretch position in a supine position with his legs extended, his arms placed along his torso and his head facing south.

Kleinaitingen (KLE, lat: 48.22 - lon: 10.84, Germany)

Contact persons: Ken Massy, Philipp W. Stockhammer

A detailed site description can be found in (26, 27). Kleinaitingen "Gewerbegebiet Nord" is located in the Lech Valley, in what now corresponds to the city of Augsburg, and was excavated between 2012 and 2014 by the local history and archeology society. It consists of 63 graves, containing mostly individual inhumation graves but also double and triple burials have been found. A total of 72 individuals were excavated, and some cremations as well as burials without bones due to disturbance have been identified. The site dates to the Early Bronze Age 2116-1514 cal BCE and with a subsistence focus on agriculture. Several animal bones, mainly cattle, were found at the site. A total of 31 individuals have been analyzed for their human DNA (27), and of those 28 individuals were screened for pathogens.

From the 28 individuals, 2 were positive for *Y. pestis*:

KLE031 is a genetically female infant (3-4 years) who was found in a triple burial containing two more individuals, all of them subadults. KLE031 (AITI_77A) and KLE032 (AITI_77B) share the same mitochondrial haplogroup (I1a1) and have been inferred to be siblings (27).

KLE048 (AITI_120) is an adult male individual (ca. 30 years), buried with a dagger, and who also is genetically related to three individuals buried close-by at the site (27).

Kaluzhnyy 1 (KLZ, lat: 44.84 - lon: 42.21, Russia)

Contact persons: Alexandra Buzhilova, Sabine Reinhold, Svend Hansen

The kurgan cemetery Kaluzhnyy-1 is located 3 km to the southeast of the village Kaluzhnyy. It was investigated in the course of rescue excavations by the heritage organization “Nasledie” in 2008. The cemetery included three barrows. Altogether, 37 burial and an unclear complex were excavated (2 Early Bronze Age; 3 Middle Bronze Age; 18 end of Middle Bronze Age Lola culture; 5 Sarmatian time, 1 modern, 8 not dateable complexes). By the start of the excavation, mound 1 was 1,44m high with a diameter of 28 m.
We screened 1 individual for the presence of *Y. pestis*:
KLZ001 (BZNK-1029/1, kurgan 1, grave 8): Single inhumation without recognizable traces of a grave-pit (set into the mound shell), in the southern part of the mound, which was disturbed during excavation of grave 7. It is associated with the end of the Middle Bronze Age Lola culture and dated in the present study to 3793 ± 25 (MAMS-45952, 4243-4091 cal BP). The skeleton is in a crouched position on the left side with the head towards northeast. Various organic remains were found around the skeleton: yellow organic remains on the lower part of the body; brown organic remains under the skeleton; charcoal and ochre around left hand and shoulder; and white organic remains to the east of the left shoulder and shinbone. Multiple grave goods were found including 41 colored faience beads, 12 mother of pearl pendants and 2 animal teeth pendants.

Krasnogvardeyskoe (KNK, lat: 45.82 - lon: 41.58, Russia)

Contact persons: Andrey B. Belinskiy, Sabine Reinhold, Svend Hansen

The burial mound of Krasnogvardeyskoye, completely excavated in 1995 (28), is located in the NW of the Stavropol region, on the left terrace of the river Yegorlyk, about 3 km east of the village under the same name. The region takes a middle position between the steppe areas on the Kuban and the central part of Stavropol region. The 2.97 high kurgan had a diameter of 60 m and consisted of at least 3 tumulus shells. Mound shell 1 consisted of light brown and gray clay, whereas mound shells 2 and 3 were made of yellow-gray clay with inclusions of yellow loam. In the kurgan a total of 23 graves, 2 ritual complexes and 2 chance finds were exposed. The oldest feature is ritual complex 2, which consists of 2 decorated vessels with similarities to Eneolithic burials of the Caucasus foreland. Possibly, they are the remains of a contemporary burial which was destroyed by grave 20. The remaining finds belong to different periods of the Bronze and Early Iron Ages. The Early Bronze Age is represented by the graves: 14, 15, 20, 21 of the Yamnaya culture, with the burials showing strong parallels to the finds of the Kuban steppe zone. Grave 8 has parallels with both the Pre-Catacomb graves and those of Novotitorovska culture. Grave 1 is assigned to the North Caucasus culture. Another group of 8 graves (2, 9, 10, 13, 16, 18, 22, 23) is associated with the Yamnaya culture. Burial 7 can be dated to the end phase of the Middle Bronze Age or the Late Bronze Age and may be connected with the spread of the Srubna culture into the Caucasus foreland. Graves 6 and 19 are attributed to the Beloserska culture. They are the easternmost known complexes of this cultural phenomenon, which otherwise spread from the northern Black Sea to the lower Danube. The graves 4 and 12 without inventory can only be

generally assigned to the Bronze Age. The remaining tombs (3, 11, 17 and probably 5) are Sarmatian.

In the present study we screened 6 individuals for the presence of *Y. pestis* DNA, and identified one positive individual:
KNK001 (BZNK-1016/1, kurgan 1, grave 8): Single inhumation associated with the Novotitorovskaya-culture located in a stepped pit imitating a wagon, located in the south-eastern sector of the burial mound, east of the central profile bar. It was heavily disturbed by bulldozers. The skeleton was found in a slightly flexed position on the right side with the head facing NW and the feet were dyed with ochre. Under and around the skeleton, a layer of dark brown organic matter was found and beneath it there was a larger layer of red-brown organic matter. A thick layer of charcoal was found around the legs. The floor beneath the skeleton appears to be burnt and the pit floor was covered with lime, with walls lined with wood, and along the SO-wall remains of organic matter with a woven pattern. A silver earring, a bronze dagger and a bracelet of 50 bronze beads were found as grave goods.

Kyzyl (KZL, lat: 48.27 - lon: 75.35, Kazakhstan)

Contact persons: Arman Beisenov, Leyla B. Djansugurova, Elmira Khussainova, Egor Kitov, Lyazzat Musralina

A more detailed site description can be found in Beisenov et al. (2016, 2018) (29, 30). This site is located in Central Kazakhstan, in the territory of the Aktogay district of the Karaganda region. It is located on the slope of Mount Begazy, on the right bank of the river Karatal. At a distance of 1.2 km to the south is the famous burial ground of Begazy.
Previously, individuals from the site were published in Gnecchi-Ruscone et al. (2021) (31). From those individuals, we screened 5 individuals for the presence of pathogen DNA, and only one was positive for plague (KZL002, mound 4, right side). AMS 14C dates of the analyzed sample (KZL002) based on Tasmolinskaya culture, 2491±33. Dimensions 2.3 × 1.28 m, tapering to the bottom. At a depth of 0.8 m, it has an oval shape and dimensions of 1.7 × 1.2 m. At this depth, two human skeletons are found, one to the left and one to the right (positive individual for *Y. pestis*, KZL002). Head is facing to the northwest, with very bent legs (half-crooked pose). Near the left bone and the left shoulder joint, as well as several dorsal vertebrae, to the left of the pelvis was a bronze mirror with a side handle with a curly end. Despite the fact that there is the possibility of filling the grave, with some indications in the left skeleton, apparently disturbed in ancient times. Stone "pillows" in the form of an oblong shape were also found in the grave.

Mikulovice (MIB, lat: 49.99 - lon: 15.77, Czech Republic)

Contact person: Michal Ernée

A more detailed site description alongside the genome-wide data from several individuals from this site is published in Papac et al. 2021 (16) and in Ernée et al. (2020) (32). The site is located in eastern Bohemia, south of Pardubice, along a small nameless watercourse. It was discovered during the construction of houses in the north of the village center and rescue excavations took

place in 2006-2012 by J. Frolík, R. Sedláček with occasional excavations until today. The site was occupied from at least the Early Bronze Age on until the Roman Iron Age. While finds from the Únětice dominate, there is also extensive evidence for the Urnfield period, the Late Hallstatt and the Roman Iron Age.

In total, 91 individuals from the Early Bronze Age were screened for the presence of pathogen DNA, of which one sample tested positive for *Y. pestis*:

MIB054 (grave 42, feature 2040, NM Prague Inv. No.: P7A 43141) was a male child of about 6.5-7.5 years. He was radiocarbon-dated to 3899-3730 cal BP (2 sigma, MAMS-30479, 3550±19 BP).

Oberottmarshausen (OOH, lat: 48.23 - lon: 10.86 Germany)

Contact persons: Ken Massy, Philipp W. Stockhammer

A detailed site description can be found in (26, 27). The Middle Bronze Age burial site of Oberottmarshausen “Kiesgrube Lauter” is located in the Lech Valley, 600 meters north from Kleinaltingen, and was part of a rescue excavation in 2004 (33). A total of 32 graves were excavated containing 35 individuals. A total of 20 individuals were analyzed for ancient human DNA (27) and all of them were screened in the present study for the presence of *Y. pestis* DNA.

OOH003 (OTTM_84, Bef. 84) is a genetically female adult individual (30-40 years) with no detected relatives at the side and buried in a stretched supine position with two ceramic vessels found in the grave (27). She was radiocarbon dated to 3586-3447 cal BP (2 sigma, MAMS-21543, 3283±32 BP).

Velešovice (VEL, lat: 49.17 - lon: 16.84, Czech Republic)

Contact persons: Jaroslav Peška

The Velešovice site was investigated as part of the rescue excavation during the construction of a motorway between 1985-1988. The site is located on a gentle slope facing south to southeast at an elevation of 225-230 m. It is defined to the south by the edge of a terrace falling into the Rakovec Creek inundation zone. A total of 16 graves were discovered in the site.
We screened 50 individuals from the site for the presence of pathogenic DNA, which resulted in 1 positive individual described below:
VEL003 (P7A 16167): is a male individual aged 15-17 associated with the Corded Ware Culture. The individual was C14-dated to 3951 ± 26 BP (MAMS-34184, 4516-4297 2 sigma cal BP). Various grave goods were found in the grave including various blades, pots as well as pins.

Vlineves (VLI, lat: 50.37 - lon: 14.44, Czech Republic)

Contact persons: Petr Limburský, Miroslav Dobeš, Petr Velemínský

This site description alongside the genome-wide data from the respective individuals is published in Papac et al. 2021 (16), for a more detailed archeological description see (34–38). During a

rescue excavation in 1999-2008 by V. Salač, I. Pleinerová, Ž. Brnić, P. Limburský, an area of 70 ha of this site was explored. It is located in central Bohemia and had been occupied from as early as the Jordanów culture until the Early Middle Ages. Burials from all major prehistoric periods and the migration period were found during the excavation.

A total number of 54 individuals were screened for the presence of pathogen DNA, out of which three were associated with the late Neolithic Jordanów culture. 35 individuals were selected from a Corded Ware cemetery which comprised 75 burials, five from a Bell beaker cemetery with 34 inhumations and eleven from the early Bronze Age Únětice culture cemetery with 304 graves. From these, one individual buried at the Corded Ware cemetery tested positive for *Y. pestis*:

VLI092 (Grave 9566A, NM Prague Inv. No.: P7A 41821) was a male of 40-60 years, radiocarbon-dated to 4832-4619 cal BP (2 sigma, MAMS-45801 4176±26 BP; also CRL-10180 (4057±87) 4831–4299 cal BP 2-sigma). He was buried on his right side in a crouched position with his head towards the west-north-west together with chipped blades as grave goods.

Methods

Data generation

A total of 252 individuals from 15 sites in Eurasia dating between ~5000-2000 years Before Present (yBP) were screened for ancient DNA evidence suggestive of the presence of *Y. pestis* (Supplementary Table 1). Laboratory work was performed at the ancient DNA lab facilities of the Max Planck Institute for the Science of Human History in Jena, the Max Planck Institute of Evolutionary Anthropology in Leipzig, and the Department of Genetics, Harvard Medical School in Boston. For samples from the Dereivka I site, DNA extraction was performed in Earth Institute and School of Archaeology, University College Dublin, Belfield, Dublin 4, Republic of Ireland, and sent to Boston for further processing, as previously described (24).

In Jena, teeth were sampled as described in (39) with modifications in the UV irradiation specified in the following sentences. In short, teeth were irradiated with UV light from two sides for 30 min each and then cut at the cemento-enamel junction. With a dentist drill, 30-60 mg of powder were drilled from the surface of the inner pulp chamber and the root canals. DNA from this powder was extracted with an established protocol (40, 41). The powder was incubated with 1 ml of extraction buffer (0.45 M EDTA, 0.25 mg/ml Proteinase K, pH 8) for at least 16 h at 37°C under rotation. After centrifugation for 2 min at 15,800 g, the supernatant was mixed with a 10 ml binding buffer (5 M guanidine hydrochloride, 40 % isopropanol) and 400 µl 3M sodium acetate. To bind the DNA, silica-based spin columns were used (High Pure Viral Nucleic Acid Large Volume Kit; Roche). After washing twice with washing buffer (High Pure Viral Nucleic Acid Large Volume Kit; Roche), DNA was eluted in two steps with 50 µl TET (10 mM Tris, 1 mM EDTA, 0.05 % Tween-20, pH 8) each to a final volume of 100 µl.

From these extracts, double-indexed double-stranded Illumina sequencing libraries were prepared (42) with initial USER enzyme (New England Biolabs (NEB) treatment to reduce aDNA damage in form of deaminated cytosines (43). A summary of the protocol used (44) follows: 25 µl of extract were mixed with 0.072 U USER Enzyme in 1 x Tango buffer (Life technologies), 1.2 mM ATP, 0.2 mg/ml BBSA, 0.4 mM dNTPs and incubated for 30 min at 37˚C and 1 min at 12˚C. The reaction was stopped by adding 0.1343 U Uracil Glycosylase Inhibitor (UGI, NEB) and another incubation for 30 min at 37˚C and 1 min at 12˚C. After adding 0.515 U of T4 Polynucleotide Kinase (NEB) and 0.085 U T4 Polymerase (NEB), the mix was incubated at 25˚C for 20 min and 12˚C for 10 min. DNA was purified with the MinElute PCR Purification Kit (Qiagen) and eluted in 20 µl elution buffer EB containing 0.05 % Tween-20 (EBT). The eluate was mixed with 0.25 µM sequencing adapters and 0.125 U Quick Ligase (NEB) in Quick Ligase buffer and incubated for 20 min at room temperature. DNA was purified as described above and eluted in 22 µl EBT. DNA was then incubated with 0.4 U Bst 2.0 Polymerase (NEB) and 0.5 mM dNTPs in Isothermal buffer (NEB) at 37˚C for 30 min and 80˚C for 10 min. All libraries were double-indexed with a unique pair of 8 nt long indices in reactions of 0.025 U Pfu Turbo Polymerase (Agilent Technologies), 100 mM dNTPs, 0.3 mg/ml BSA, and 0.2 µM of indices in Pfu Turbo buffer in a thermocycler with the following program: 2 min at 95˚C, ten cycles of 30 sec at 95˚C, 30 sec at 58˚C and 1 min at 72˚C, and a final elongation with 10 min at 72˚C.

The laboratory process for the archeological sites of Dereivka I and dolmen “El Sotillo” have been previously described in (23, 24) respectively.
All libraries were shotgun sequenced to 5 million reads on an Illumina HiSeq 4000 with a single-end kit (1 x 76+8+8 cycles) in Jena or a NextSeq500 with a pairer-end kit (2 x 76+7+7 cycles) in Boston and screened for the presence of pathogen DNA as described below.
For VEL003 and GRH001 single-stranded libraries were prepared for in-solution from 30 µl DNA extract with an automated protocol (45). Additional single-stranded libraries were also prepared for OOH003, KNK001, KLZ001 and ARS007 following the aforementioned protocol and enriched for *Y. pestis* DNA with in-solution capture as explained above. All single-stranded libraries were prepared in the Jena laboratories with the exception of OOH003 and GRH001 which were prepared at the Leipzig facility.

Pathogen Screening

Pre-processed reads from shotgun sequencing data (252 samples in total) were screened for the presence of pathogen DNA using the screening pipeline HOPS v.0.35 (46). In the first step, adapter-clipped reads were used as input for the MEGAN Alignment Tool (MALT, v.0.4.0, (47)) and mapped against a custom RefSeq Genome database comprising all complete viral and bacterial genomes (as of 2017), a selection of eukaryotic pathogen genomes and the human reference sequence GRCHh38. Mapping parameters were set to a minimum of 90 % identity (--minPercentIdentity) and top percent value (--topPercent) as well as minimum support (--minSupport) to 1 with BlastN mode and semi-global alignment type. All other parameters were used in default settings. The output was then filtered as implemented in MaltExtract of HOPS with a predefined list of pathogens, and assigned reads were evaluated based on aDNA damage patterns and their edit distance to the reference genomes. Additionally, MALT mapping results were visually inspected in MEtaGenome Analyzer (MEGAN, (48)).

Y. pestis enrichment

Libraries putatively positive for *Y. pestis* DNA were amplified to a concentration of 200-400 ng/µl with IS5/IS6 primers and enriched for *Y. pestis* DNA with in-solution whole genome capture as described before (49). The probe set was designed with a combination of *Y. pestis* genomes including *Y. pestis* CO92 chromosome (NC_003143.1), CO92 plasmid pMT1 (NC_003134.1), CO92 plasmid pCD1 (NC_003131.1), KIM 10 chromosome (NC_004088.1), Pestoides F chromosome (NC_009381.1) and *Y. pseudotuberculosis* IP 32952 chromosome (NC_006155.1) as a template. The capture was performed on 96-well plates in two rounds.
After capture, samples were sequenced on an Illumina HiSeq 4000 platform with a 75 bp paired-end kit (2 x 76+8+8 cycles; samples ARS007, GRS004, HOP001, HOP004, KNK001, MIB054, I5884, I2470, KZL002, KLZ001) and/or a 75 bp single-end kit (sample KLZ001, OOH003ss, GRH001, VEL003). Additionally, the KLE031, KLE048, OOH003 samples were sequenced with a 75bp single-end kit and MIB054 with 75 bp paired-end on an Illumina NextSeq500.

Data processing

Raw data was processed with nf-core/eager (v2.2.2 (50)), with the exception of I5884 and I2470 samples that required a preprocessing step to remove the 7bp internal barcodes. These samples were preprocessed as follows: we ran AdapterRemoval v.2.3.1 (51) to clip only adapters and extracted reads containing the barcodes using grep (v.3.1, http://www.gnu.org/software/grep/). We ran the script fastq_trimming_barcodes.sh, which removed the barcodes by trimming 7 bp from each end using FASTX-trimmer v.0.0.14 (52), and removed the reads without a pair (one of reads of the pair did not contain a barcode) with filterbyreadname.sh from BBmap from the tool suite BBTools (53). The fastq files after removal of the barcodes were uploaded in the read repository. The preprocessed read pairs for the libraries were listed in a tab separated value (tsv) file used as input for nf-core/eager together with the rest of the raw data. nf-core/eager was run with the tsv input and the following processes were run: FASTQC v0.11.4 (54) was run to evaluate the quality of the sequencing data. Adapter clipping, filtering of short (<30bp) or low-quality reads and merging of pair-end data were performed with AdapterRemoval. Prior to mapping, reads from the same libraries were merged. We mapped the reads against the *Y. pestis* CO92 reference (NC_003143.1) with bwa aln v.0.7.12 (55) adapting the seed length (-l) to 16 and the mismatch allowance (-n) to 0.01, in order to increase the sensitivity of the mapping that can be compromised due to the highly fragmented nature of ancient DNA. To retain reads mapping uniquely to the reference, we filtered reads with mapping quality lower than 37 with SAMtools v1.3 (56). We then removed duplicates with Picard Tools v1.140 MarkDuplicates (57), merged different libraries from the same individual, and calculated the mapping statistics with Qualimap v2.2.1 (58,59). To authenticate the ancient origin of the molecules characterized by deamination of C → T dur to hydrolytic damage we calculated deamination patterns with DamageProfiles v0.4.9 (60). To remove potential bias introduced due to the aforementioned deamination, we removed the damaged bases before further analysis. This was achieved by removing 1 bp from each end of the reads using FASTX-trimmer, except for the single-stranded libraries where no trimming nor additional mapping was applied. The resulting trimmed fastq files were processed as before with exception of the skipping of AdapterRemoval and setting the bwa aln parameters to -n 0.1 and -l 32 to be stricter during the mapping step. As before, duplicates were removed with Picard Tools MarkDuplicates. Libraries produced from the same individual were combined after the removal of duplicates independently and mapping statistics were then calculated with Qualimap as described above. The obtained bam files were then realigned with GATK v3.5 realigner and vcf files were obtained using GATK UnifiedGenotyper (61).

To explore the presence of the *Y. pestis* plasmids in the ancient samples, we repeated the mapping steps by independently mapping the preprocessed reads to the pCD1 (NC_003131.1), pMT1 (NC_003134.1) and pPCP1 (NC_003132.1) CO92 plasmids.

Variant calling, SNP effect and phylogenetic analysis

To produce the final SNP alignment containing all the variable positions to be used in the phylogenetic analysis, we ran MultiVCFAnalyzer (62) (https://github.com/alexherbig/MultiVCFAnalyzer) with the vcf files for the ancient genomes from this study but also including previous ancient samples from the first pandemic (63, 64), second pandemic (65–68), prehistoric genomes (10, 49, 69–72) and modern genomes (73–80, 80–84)

summarized in Dataset S2. In order for a SNP to be called, it must fulfill the following criteria: the allele must be supported by 90% of the reads, with a minimum of 4 reads supporting the call. For the single-stranded libraries an additional genotyping was performed, utilizing the characteristic of the library construction for which we only have C → T observable damage. Here, damage will be observed as C → T change in forward mapping reads and as G → A in the reverse mapping reads. We split the realigned bam file into forward and reverse mapping reads with samtools view, which were then run together with the complete dataset in MultiVCFAnalyzer with the same parameters as above. We implemented the publicly available genoSL.R script (https://github.com/aidaanva/GenoSL) for the purpose of genotyping single-stranded libraries as follows: for all substitutions to T calls were drawn based on the reverse mapping reads in that position, for all A substitutions calls were drawn from the forward mapping reads and for all other substitution the call was drawn from the complete dataset. The resulting SNP Table and a corrected SNP alignment was then used for further processing.

Previous studies have shown that an abundance of environmental background in metagenomic datasets can result in the incorporation of false SNP calls during ancient bacterial genome reconstruction (67). Most often, such erroneous calls manifest themselves in a phylogenetic analysis as private SNPs, as they are unlikely to be shared with the ingroup diversity (63, 67). As a consequence, erroneously calculated private branch lengths can interfere with evolutionary inferences and divergence date estimates (67). Here, in order to filter out private SNP calls that result from environmental contaminants, we used the SNP Evaluation (63) (https://github.com/andreasKroepelin/SNP_Evaluation) to co-analyzed all newly reported genomes in this study as well as previously published genomes RISE509, RISE505 (10), Gyvakarai1, KunilaII, 6Post, 1343UnTal85, GEN72, RK1001 (49), GZL001 and GZL002 (72). SNP calls were evaluated on the basis of sample laboratory processing, where UDG-treated data were assessed under different criteria from non-UDG-treated data to account for substitutions associated with aDNA damage in the latter.

For UDG-treated genomes, unique SNPs were assessed within a 50 bp window and were accepted as “TRUE” when:

1. A comparison between lenient mapping (BWA parameters -n 0.01, -l 16) and stringent mapping (BWA parameters -n 0.1, -l 32) resulted in <10% coverage increase around each SNP.
2. No heterozygous SNP positions were identified within the evaluated region (50 bp window) around each private SNP.
3. No gaps in genomic coverage were observed within the evaluated region around each private SNP.

For non-UDG-treated genomes, unique SNPs were assessed within a 50 bp window using only lenient mapping parameters (BWA setting -n 0.01, -l 16) and were accepted as “TRUE” when:

1. The evaluated SNP position was not confined by aDNA damage.
2. Heterozygous SNP positions within the evaluated region (50 bp window) are only permitted when consistent with aDNA damage (C-to-T or G-to-A substitutions).
3. No gaps in genomic coverage were observed within the evaluated region around each private SNP.

The script DeDamagevcf.R was used to remove all potential substitution due to damage from nonUDG vcfs files, which was then used as input for SNP Evaluation to count the heterozygous

calls. The unmodified vcfs were used to count non covered positions for nonUDG samples, and to count non covered positions as well as heterozygous calls in UDG-treated genomes. All the positions assessed can be found in Dataset S11, S12, S13. The false-positive SNPs were excluded with MultiVCFAnalyzer, together with previously identified homoplastic regions (See https://github.com/aidaanva/LNBAplague/blob/main/multiVCFAnalyzer/SNPstoExclude) and genoSL.R was run to obtain the final SNP table and final SNP alignment containing all the variant sites in the dataset analyzed. The effect of SNPs specific to the LNBA- lineage was analyzed with SnpEff v3.1 (85). The snpTableForSnpEff.tsv output from MultiVCFAnalyzer was used as input for SnpEff with a prebuilt SnpEff database based on the *Y. pestis* reference genome CO92 (NC_003143.1). The resulting annotated file was included in a second run of MultiVCFAnalyzer and genoSL.R as described above to obtain a snpTable annotated with the effects which was then filtered for SNPs found exclusively in the LNBA- lineage. For pseudogene analysis, only SNPs resulting in the gain or loss of stop and start codons were considered. The loss of stop codons in genes present as pseudogenes in the reference genome CO92 were not considered as pseudogenization since we assumed that the gene was not yet pseudogenized on the LNBA- branch. All SNPs leading to pseudogenization which were present in at least one strain of the LNBA- branch but were called as N by MultiVCFAnalyzer due to low coverage or possible aDNA damage were inspected in IGV (86). If the position of these SNPs was covered by at least one read with the corresponding SNP present in the read(s), it was counted as “low coverage”. Gene *flgB*, which became non-functional through the gain of a stop codon, was then lost completely as part of a larger genomic region in the youngest strains and therefore also counted as “called”. Only genes on the chromosome were considered for this analysis. Finally, the SNP alignment was used to compute a Maximum Likelihood tree with RAxML-NG (v. 0.9.0, https://github.com/amkozlov/raxml-ng) with the following command:

```
raxml-ng --all --msa $CurrentAlignment --model GTR+G --seed 2 --threads 9 --bs-trees autoMRE --
prefix $name
```

where $CurrentAlignment is the fasta alignment and $name is the output prefix for the current run.

Bayesian molecular dating of I5884

Given the incongruence between the phylogenetic positioning and radiocarbon date of I5884 (Figure 1B and C), we applied a molecular dating approach using the program BEAST v.2.6.6 (87) to re-evaluate the specimen’s age. For this, we initially used the program TempEst v1.5 (88) (http://tree.bio.ed.ac.uk/software/tempest/) to assess the temporal signal across the LNBA- lineage, using all previously published and newly available genomes as well as their associated calibrated median radiocarbon dates (see Supplementary Figure 2). Overlapping variant positions across all LNBA- isolates and the modern branch 0 strain 0.PE2 Pestoides F (used as outgroup) were used for the construction of a maximum parsimony tree (using all data - 2,037 variants) in MEGA7 (89), which was used as input for TempEst in NEXUS format. Our analysis revealed a near perfect correlation between specimen ages and their tip distances from the root for the LNBA- lineage ($R^2=0.97$), which permitted us to pursue a tip dating analysis for I5884. Subsequently, the same SNP dataset was used as input for BEAST v2.6.6, including all available calibrated radiocarbon age ranges in years BP as uniform priors and the age of the 0.PE2 isolate set to 0 (See https://github.com/aidaanva/LNBAplague/blob/main/Data/2020-07-

09_LNBA_leprosy_enterica_comp/LNBA_transect/Metadata_coordinates_dating_sex_updated_def.csv). Instead, the uniform prior used for the age of I5884 was set to span the entire currently known temporal range of the LNBA- lineage, between 5000- and 2500-years BP. We used BEAUti v2.6.6 to set up two separate analyses using the coalescent constant size and coalescent skyline tree priors, both with a lognormal relaxed clock and a GTR substitution model (four gamma rate categories). For each of the two analyses, independent chains of a maximum 150,000,000 states were run in BEAST v2.6.6. After completion, all runs were inspected in Tracer v1.6 (http://tree.bio.ed.ac.uk/software/tracer/) to ensure that the effective sample sizes (ESS) of all computed parameters are > 200. Subsequently, the three chains were combined using LogCombiner with a 10% burn-in. Evaluation of the posterior date of I5884 in Tracer v1.6 revealed overlapping age ranges between both computed analyses spanning from 4584 to 4406 years BP, which is in line with the genome's phylogenetic positioning.

Molecular dating analysis

In order to estimate the divergence timing between the LNBA- clade and all other *Y. pestis* diversity, we used the Bayesian phylogenetic framework BEAST v2.6.6 (87). For this, we compiled a dataset including all described LNBA- strains with >3-fold average coverage, the previously described RV2039 genome isolated from a ~5,000-year-old hunter-gatherer, and a subset of genomes representing all *Y. pestis* clades described to date (genome selection as in (90)). We investigated the temporal signal across these data by correlating the root-to-tip distance of all strains with their associated ages using the program TempEst v1.5.3(88). For this, we used a SNP alignment of all genomes to construct a maximum likelihood tree using all variants in MEGA7 (89). The resulting tree was used as input for TempEst in NEXUS format together with the mean radiocarbon/archeological dates of ancient stains within our dataset (See https://github.com/aidaanva/LNBAplague/blob/main/Data/Radiocarbon_dates/Metadata_coordinates_dating_sex.csv). The dates of all modern strains were set to 0 years BP. The root-to-tip regression analysis showed an acceptable correlation between specimen age and distance from the tree root for the entire dataset (r=0.44). As such, we opted for a molecular dating analysis.

For the molecular dating analysis, we used an alignment of all variants (3595 SNPs) as input for the set-up in BEAUti, including mean radiocarbon dates of all the ancient strains as starting tip dates in years before the present (yBP) and the ages of all modern strains set to 0 yBP. In addition, the posterior ages of all ancient strains were estimated based on uniform prior distributions that were constrained according to each specimen's two-sigma (95.4%) calibrated radiocarbon age interval or on their archeological contexts (See https://github.com/aidaanva/LNBAplague/blob/main/Data/2020-07-09_LNBA_leprosy_enterica_comp/LNBA_transect/Metadata_coordinates_dating_sex_updated_def.csv). For I5884, whose specimen's radiocarbon date was found to be inconsistent with the *Y. pestis* genome's phylogenetic positioning, we used a uniform age prior spanning the entire temporal breadth of the LNBA- lineage, ranging from 5000 to 2500 BP. The entire *Y. pestis* clade excluding RV2039 as well as the LNBA- lineage were constrained as monophyletic clades for the analysis. Moreover, we tested two model settings using the constant coalescent and the coalescent skyline demographic models, in combination with a lognormal relaxed clock and the GTR model of nucleotide substitution (four gamma rate categories), as previously described (90). We used path sampling (PS) implemented in BEAST v2.6.6 to evaluate the suitability of each

tested model for the present dataset. PS was run for 50 independent steps of 20 million states each and produced marginal likelihood estimates of -24677.5536 and -24656.1318 for the coalescent constant size and coalescent skyline models, respectively. After comparing the marginal likelihood estimates of both models, the resulting log Bayes factor (21.4) strongly favored the coalescent skyline model (91). Therefore, this model was used for all subsequent molecular dating analyses. We run two independent chains of up to 300,000,000 states, with a sampling frequency every 10,000 states. Furthermore, after completion of each analysis, individual chains were combined using LogCombiner, with 10% burn-in, and the results were viewed in Tracer v1.6 (http://tree.bio.ed.ac.uk/software/tracer/) for an evaluation of the effective samples sizes (ESS) of all posterior estimates. Run convergence was assessed for each of the individual analyses ensuring that all ESS values > 200. A maximum clade credibility (MCC) tree was created using TreeAnnotator with a 10% burn-in. The resulting MCC tree was visualized and edited in FigTree v 1.4.4 (https://github.com/rambaut/figtree/releases/tag/v1.4.4).

Virulence analysis and indel analysis

To assess the presence and absence of known virulence factors in *Y. pestis*, we compiled a bed file containing the coordinates for genes on the chromosome (n=115), and the pCD1 (n=37), pMT1 (n=6), and pPCP1 (n=1) plasmids of *Y. pestis* CO92 (Dataset S3). In order to account for regions that may have mappability issues (e.g. duplicated regions), we mapped the trimmed reads and the sslib reads as above with the exception that no mapping quality filter was applied (--bam_mapping_quality_threshold 0). The output bam files were then used to calculate the percent of the gene covered using bedtools v2.25.0 (92) and prepared the data for R using Generate_bed_files.sh. The resulting bed files were concatenated together using the cat command and the final files can be found in https://github.com/aidaanva/LNBAplague/tree/main/Data/Virulence. The results were plotted in R (93) using the ggplot2 package (94).

Additionally, we used the resulting non-filtered bam files to explore the presence of chromosomal deletions using *Y. pestis* CO92 as reference. We recovered non-covered regions from bam files as follows: bedtools genomecov was used to calculate the non-covered regions per sample. Non-covered regions separated by less than 100 bp were then merged together and subsequently filtered to have a minimum size of 500bp. We also calculated the percentage of coverage for each missing window to account for sparse data in low coverage genomes. The resulting files per sample were then combined and analyzed with R. Additionally, we extracted the genes affected by any deletion. All these steps were implemented in the script IndelCheck.sh. For the missing regions, we plotted deleted regions containing less than 15% of the region covered using the ggplot2 and ggalt (95) packages.

Phylogeography and temporal testing

To test whether the genomes in the LNBA- lineage are indeed descendants of one another, we tested whether there is a linear correlation between either genetic and geographical distance or genetic and temporal distance. We performed this analysis in R by calculating the genetic distance as the pairwise distance using the dist.dna function of the ape package (96) and as input the filtered snpAlignment.fasta from MultiVCFAnalyzer to contain only the LNBA- genomes and

their variable sites. The geographic coordinates were collected from each of the archeological sites used in this study (https://github.com/aidaanva/LNBAplague/blob/main/Data/2020-07-09_LNBA_leprosy_enterica_comp/LNBA_transect/Metadata_coordinates_dating_sex_updated_def.csv) and pairwise linear distances were calculated as the shortest distance between two geographical points the most direct path without taking in account geography as the crow flies using the distm function of the geosphere package (97). Finally, the median years Before Present (yBP) radiocarbon date was used to calculate the temporal pairwise distances using the outer function from base R. We performed a mantel statistic to test whether there was a correlation between genetic versus geographic distance matrices or genetic versus temporal distance matrices. This was performed using the mantel function from the vegan (98) package in R. A linear model was fit for either genetic versus geographic distance or genetic versus temporal distance using the lm function in R. The correlations were plotted using ggplot2.

In order to provide comparative data for these correlations, we performed the same analysis using high coverage genomes from the second plague pandemics, ancient leprosy (*M. leprae*) and ancient *Salmonella* data (Supplementary Table 5, see https://github.com/aidaanva/LNBAplague/tree/main/Data/2020-07-09_LNBA_leprosy_enterica_comp subfolders for the data). The final figure was generated in R using the ggpubr package (99).

All the previously described R code can be found in the R notebook here: https://github.com/aidaanva/LNBAplague/blob/main/Stone_Age_Plague_v5.Rmd.

Supplementary Figures

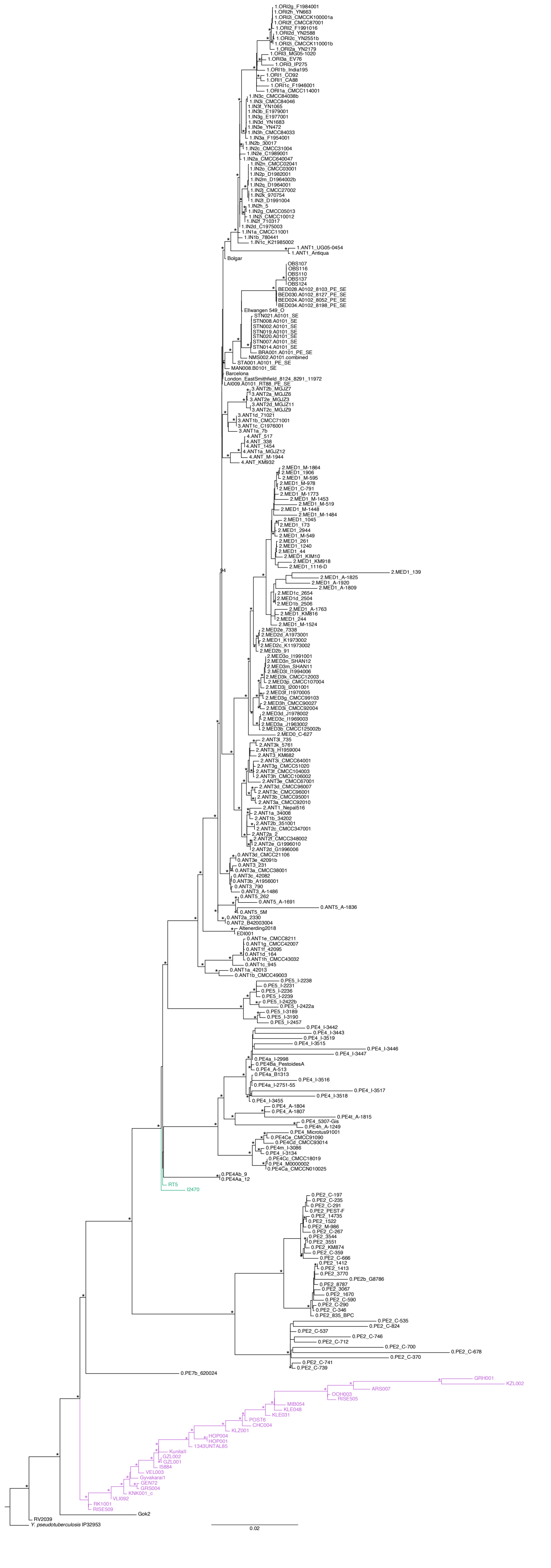

Supplementary Figure 1: **Maximum Likelihood tree for ancient and modern *Y. pestis*.** The tree was computed from a SNP alignment (n=7,506) using RaxML-ng and 1000 bootstraps replicates. Bootstrapping values above 90% are indicated as an asterix in each node. Colors indicate specific lineages being purple the LNBA- lineage and green LNBA+ lineages. The tree was plotted with FigTreev1.4.4 (https://github.com/rambaut/figtree/releases/tag/v1.4.4). Related to Figure 1B.

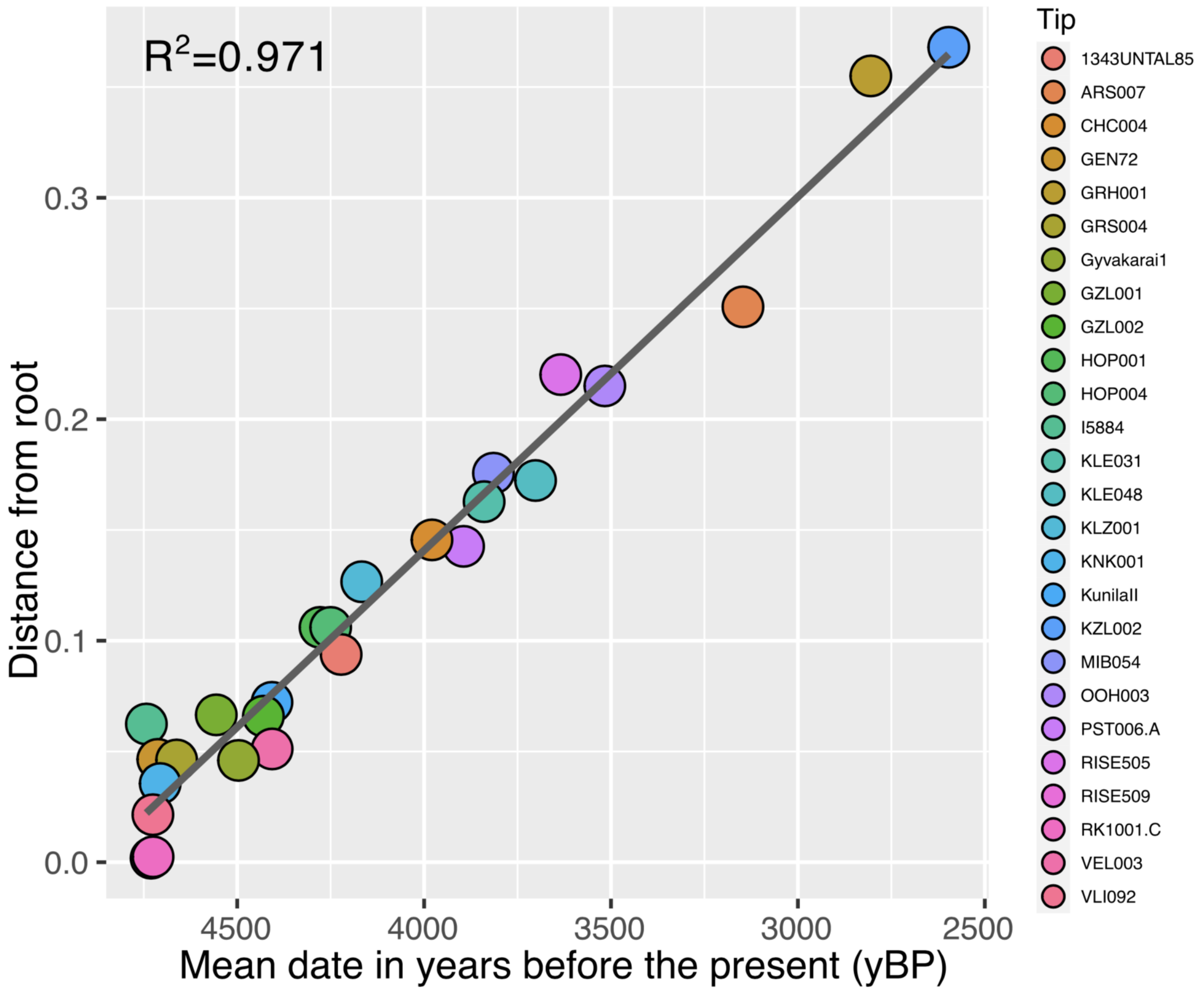

Supplementary Figure 2: **Root to tip regression analysis for the LNBA- lineage.** The analysis was performed with TempEst and visualized with ggplot2 (94) and R v3.6 (93).

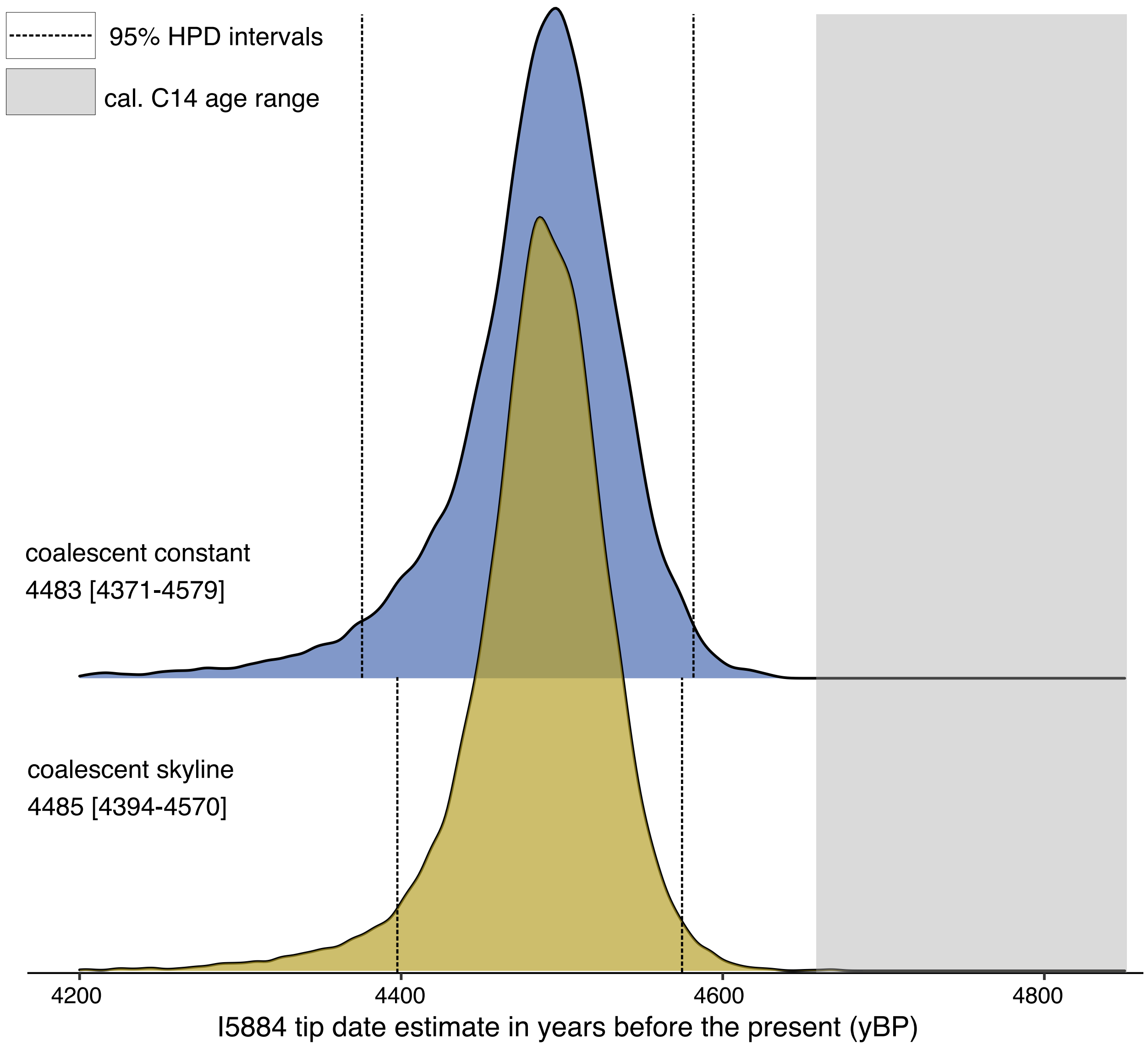

Supplementary Figure 3: **Distribution of the posterior probabilities of the inferred tip date of I5884 by BEAST v2.6.6**. The colors indicate the demographic model used, yellow being coalescent skyline and blue coalescent constant. The plot was generated using the ggplot2 (94) package within R v3.6 (93).

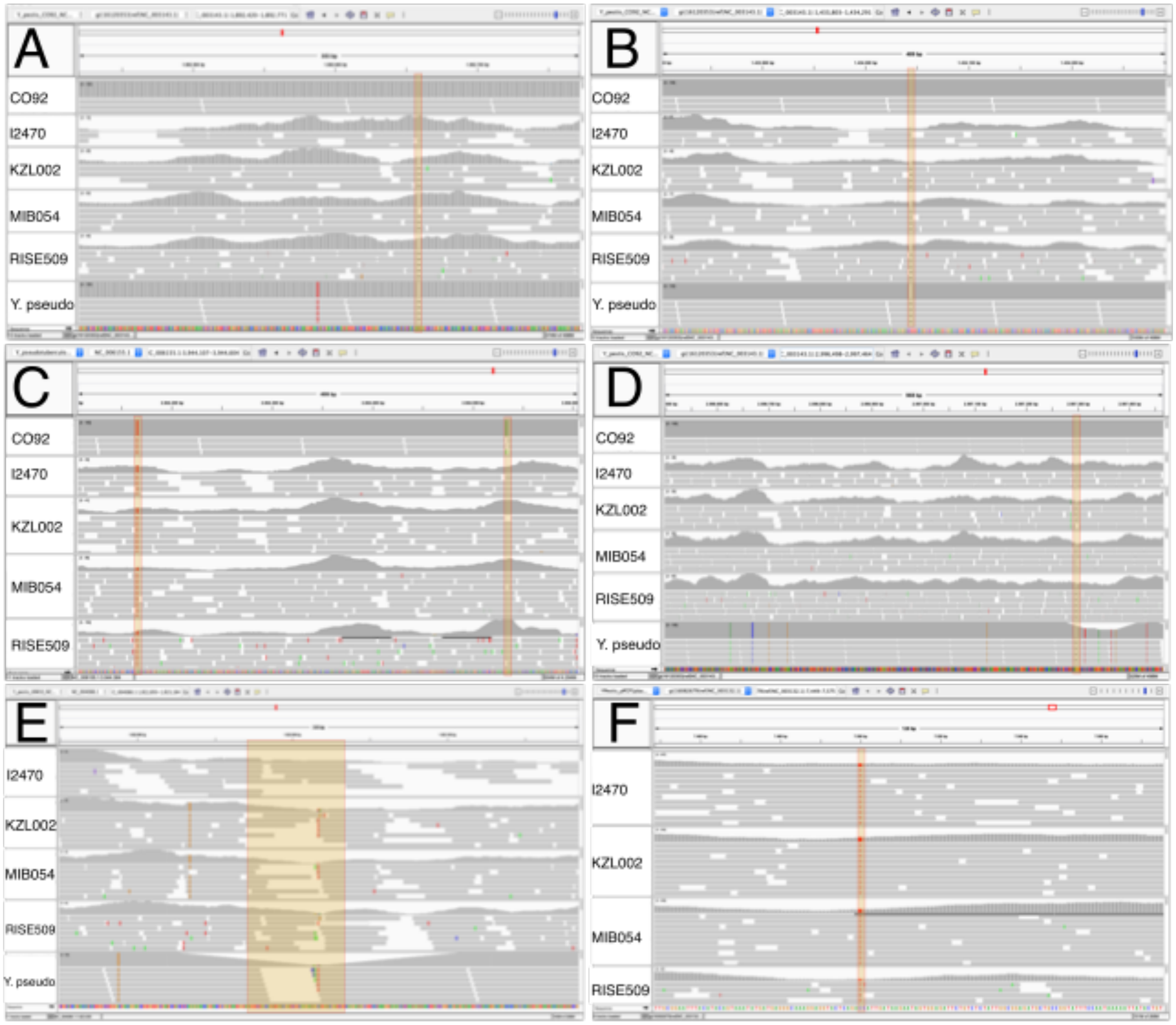

Supplementary Figure 4: **Manual evaluation in IGV[91] of genes involved in flea transmission and virulence in the I2470, KZL002, MIB054, RISE509 and compared with *Y. pestis* CO92 or *Y. pseudotuberculosis* (Y. pseudo):** A) *flhD*, B) *pde2*, C) *pde3*, D) *ureD*: , E) *rcsA*, F) *pla* gene were we observe the 'T' variant in all prehistoric strains in 7,500 position of the pPCP1 plasmid, which corresponds to the ancestral variant that results in the isoleucine amino acid in the position 259 of *pla* (100). For *flhD, pde2, ureD* and *rcsA*, we observe the active form the LNBA- lineage strains while I2470 presents the inactive form. For *pde3*, an intermediate state is found in all the prehistoric genomes where one of the mutations (T, form PDE-3-*pe'*) has already been gained by all the prehistoric strains but the other has still not been acquired (A mutation).

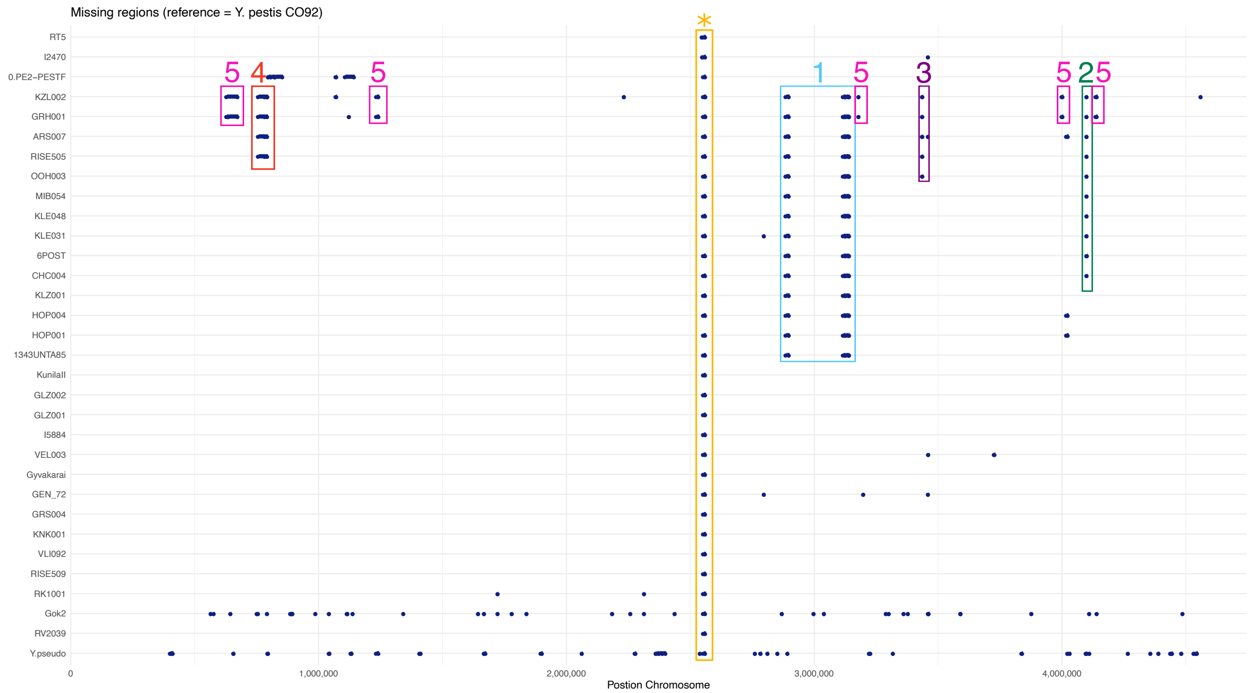

Supplementary Figure 5: **Missing region evaluation for the prehistoric *Y. pestis* genomes.** Dumbbell plot showing missing regions of at least 500bp detected in this study's dataset when using *Y. pestis* CO92 as reference. The x axis represents positions in the chromosome of the reference genome and strains are ordered on the y axis based on their phylogenetic placement. The numbered squares indicate the deletions in the LNBA- lineage, and the numbers correspond to the ones displayed in Figure 1B, and Dataset S4. * represents the filamentous prophage YpfΦ, which is only consistently integrated in the chromosome of 1.ORI strains. Gok2 presents a high number of deletions, however this is probably due to the low coverage of this sample. Deletions found in *Y. pseudotuberculosis* indicate regions not present in the ancestor. The plot was generated with ggalt (95) and ggplot2 (94) packages using R v3.6 (93).

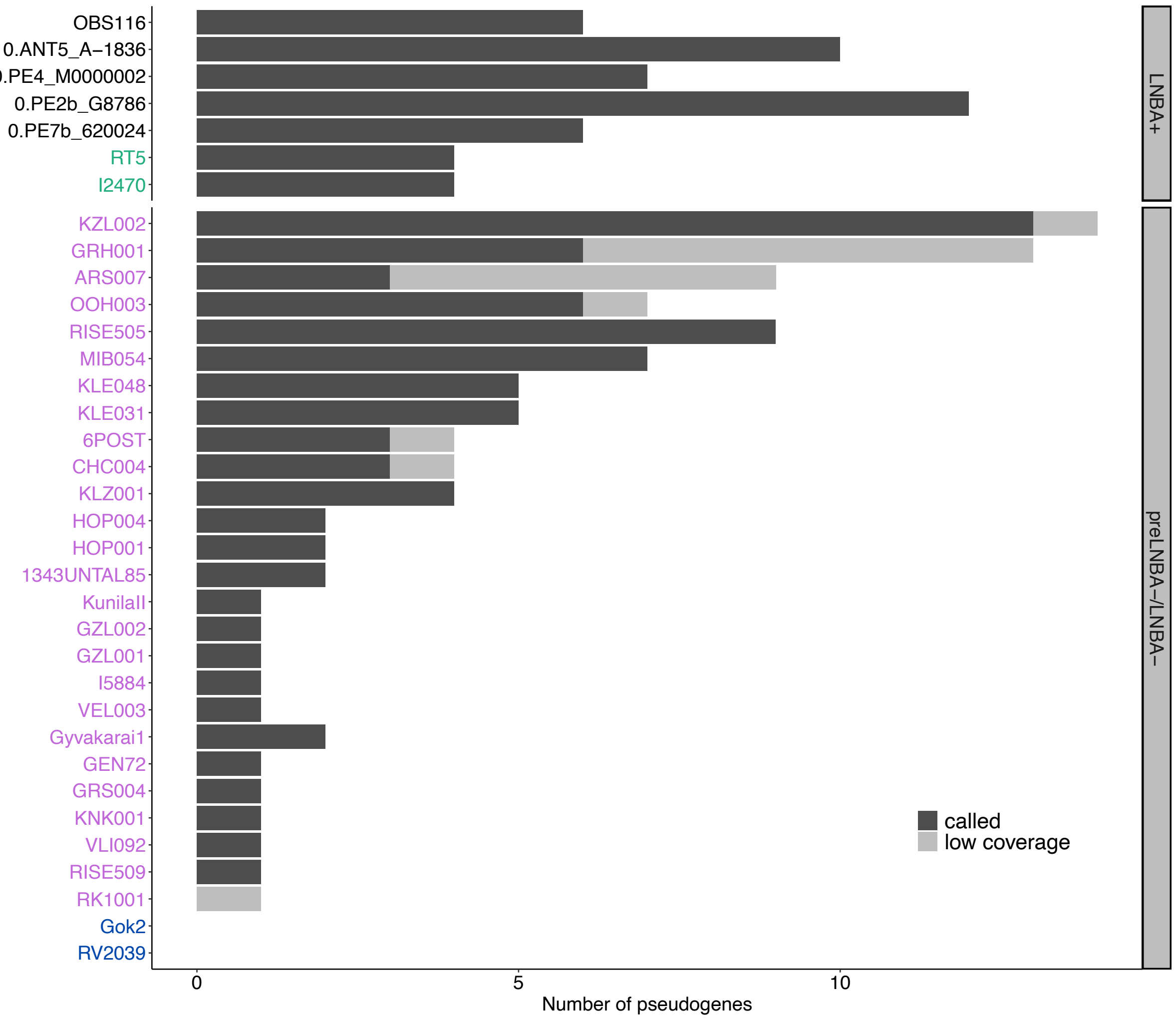

Supplementary Figure 6: **Count plot of pseudogenes on the chromosome for different *Y. pestis* strains**. This includes the genomes of the LNBA- (purple) branch, which are compared to the preLNBA- (blue) and LNBA+ (green) strains as well as extant representatives of branches 0.PE7, 0.PE2, 0.PE4, 0.ANT5 and the second pandemic genome OBS116 based on SNP effect analysis (Dataset S10). SNPs resulting in the gain or loss of a stop codon or the loss of a start codon which were called by snpEff v3.1 (85) are colored in dark gray. If these SNPs were covered and present in younger LNBA- strains but not called by snpEff due to low coverage or because of possible aDNA damage they are represented in light gray.

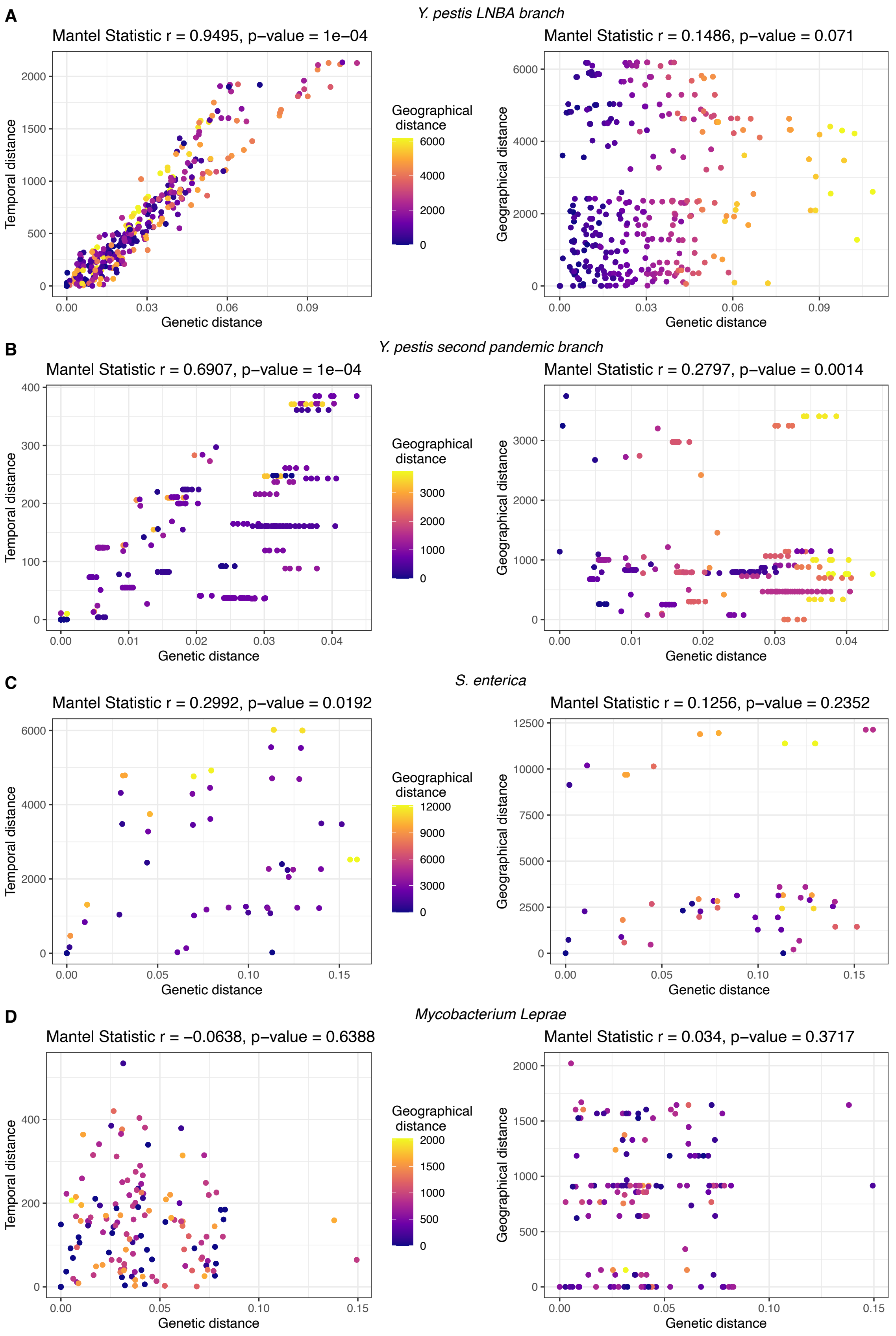
A
Y. pestis LNBA branch
Mantel Statistic r = 0.9495, p–value = 1e–04
Mantel Statistic r = 0.1486, p–value = 0.071
B
Y. pestis second pandemic branch
Mantel Statistic r = 0.6907, p–value = 1e–04
Mantel Statistic r = 0.2797, p–value = 0.0014
C
S. enterica
Mantel Statistic r = 0.2992, p–value = 0.0192
Mantel Statistic r = 0.1256, p–value = 0.2352
D
Mycobacterium Leprae
Mantel Statistic r = –0.0638, p–value = 0.6388
Mantel Statistic r = 0.034, p–value = 0.3717
Temporal distance
Geographical distance
Genetic distance

Supplementary Figure 7: **Exploring genetic, temporal and geographical distance correlations in ancient pathogens.** Correlations between temporal (years) and genetic distance with colors indicating the geographical distance (km) (left-hand side), and geographical and genetic distance with colors indicating temporal distance (right-hand side) for A) LNBA- genomes (n=26), B) Second pandemic genomes (n=27), C) *Salmonella enterica* ancient genomes (n=11), and D) *Mycobacterium leprae* ancient genomes (n=17). Each dot represents the pairwise distance between two samples. Mantel statistics were calculated using the vegan (98) package in R v.3.6 (93). Distance matrices were plotted using ggplot2 (94) and ggpubr (99) packages in R.

Supplementary Tables

Supplementary Table 1: Overview of archeological sites and number of individuals per site screened in the course of this study. Abbr. = Abbreviation for the site name; the approximate coordinates of the sites are provided in Lat. = Latitude, Lon.= Longitude

Site	Abbr.	Lat.	Lon.	Country	Number of Screened Individuals	Number of recovered *Y. pestis genomes*	Publication
Dereivka I	DER	48.91	33.76	Ukraine	20	1	(24)
Vlineves	VLI	50.37	14.44	Czech Republic	50	1	This study
Krasnogvardeyskoe	KNK	45.82	41.58	Russia	6	1	This study
Großstorkwitz	GRS	51.18	12.26	Germany	4	1	This study
Hostivice-Palouky	HOP	50.08	14.28	Czech Republic	4	2	This study
Kaluzhnyy 1	KLZ	44.84	42.21	Russia	1	1	This study
Chociwel	CHC	50.79	17.09	Poland	6	1	(10, 12), this study*
Kleinaitingen	KLE	48.21	10.84	Germany	28	2	(27)
Mikulovice	MIB	49.99	15.77	Czech Republic	91	1	This study
Oberottmarshausen	OOH	48.23	10.86	Germany	20	1	(27)
Dolmen “El Sotillo”	ESO	42.57	-2.62	Spain	2	1	(23)
Arbulag sum, Khövsgöl	ARS	49.65	99.72	Mongolia	11	1	(4)
Kyzyl	KZL	48.27	75.35	Kazakhstan	5	1	This study
Velešovice	VEL	49.17	16.84	Czech Republic	3	1	This study
Grushevskoe	GRH	45.00	41.54	Russia	1	1	This study
**Total**					252	17	

*New data generated, including RISE139.

Supplementary Table 2: Metadata and summary statistics for the *Y. pestis* chromosome reconstruction. * new data for the previously published genome RISE139, which is referred to as CHC004 in this study. calBP= calibrated Before Present

Sample name	Arch ID	Site name	Country	AMS Lab ID	C14 (BP)	C14 dating (2σ calBP)	Mean Coverage	Coverage >4X (%)
**I5884**	I5884/Mos44, Grave 68, 294	Dereivka I	Ukraine	PSUAMS-2828	4195±20	4840-4646	11	83.87
**VLI092**	P7A 41821	Vlineves	Czech Republic	MAMS-45801	4176±26	4832-4619	17.6	91.4
**KNK001**	BZNK-1016/1, kurgan 1, grave 8	Krasnogvardeyskoe	Russia	MAMS-45959	4158±26	4827-4585	11.9	87.61
**GRS004**	PEG-07/499	Großstorkwitz	Germany	Hd-21977	4093±17	4800-4524	15.6	89.77
**HOP001**	Obj. 688 / Gr. 17	Hostivice-Palouky	Czech Republic	MAMS-30798	3837±24	4405-4152	19	91.16
**HOP004**	Obj. 691 / Gr. 22	Hostivice-Palouky	Czech Republic	MAMS-38921	3826±27	4400-4100	25	91.81
**KLZ001**	BZNK-1029/1, kurgan 1, grave 8	Kaluzhnyy 1	Russia	MAMS-45952	3793±25	4243-4091	12.6	87.02
**CHC004***	20	Chociwel	Poland	Ua-44034	3645±33	4085-3873	8	81.81
**KLE031**	Bef.77A	Kleinaitingen	Germany	MAMS-21584	3548±34	3959-3720	13.8	85.45
**MIB054**	42 (2040) NM Prague 43141	Mikulovice (big)	Czech Republic	MAMS-30479	3550±19	3899-3730	30.6	92.36
**KLE048**	Bef. 120	Kleinaitingen	Germany	MAMS-21595	3417±27	3818-3586	19.9	89.13
**OOH003**	Bef. 84	Oberottmarshausen	Germany	MAMS-21543	3283±32	3586-3447	17.4	90.86
**I2470**	I2470/ES.3/4-1	El Sotillo	Spain	Beta-299307	3060±30	3361-3181	11.5	84.16
**ARS007**	2007-9	Arbulag sum	Mongolia	NSF-Arizona	2991±48	3288-3006	7.5	68.63
**KZL002**	Kyzyl (mound 4, right)	Kyzyl	Kazakhstan	UBA-25474	2491±33	2736-2457	17	87.66
**VEL003**	P7A 16167	Velešovice	Czech Republic	MAMS-34184	3951±26	4516-4297	8.1	77.58
**GRH001**	grushevskogo pogr. 13	Grushevskoe	Russia	MAMS-47775	2707±20	2849-2761	7.6	72.34

Supplementary Table 3: Highest posterior density date intervals obtained from Bayesian phylogenetic analysis using the coalescent constant size and coalescent skyline demographic models.

Node	Coalescent skyline (95% HPD Interval)
Root (TMRCA)	5122-6174
LNBA-	4884-5360
0.PE7	3927-4718
0.PE2	3792-4185
I5884	3730-3957
RT5	3723-3921
0.PE4	3567-3954
0.PE5	2311-3860

Supplementary Table 4: Coordinates on the *Y. pestis* CO92 genome and total size of the missing regions in the LNBA- lineage. Note: regions were defined based on the largest window possible.

Event Number	Start	End	Size (bp)	IS element upstream	IS element downstream
1	2,887,052	2,900,747	13,695	Yes	No
	3,118,458	3,128,505	10,047	Yes	Yes
	3,129,749	3,140,505	10,756	Yes	Yes
	3,142,353	3,144,190	1,837	Yes	No
2	4,100,846	4,102,576	1,730	No	No
3	3,437,800	3,439,880	2,080	No	No
4	758,137	785,318	27,181	Yes	Yes
	785,985	795,686	9,701	Yes	No
5	630,297	675,989	45,692	No	No
	1,235,041	1,244,868	9,827	Yes	Yes
	3,181,310	3,182,330	1,020	No	No
	4,000,962	4,005,266	4,304	Yes	No
	4,137,897	4,143,345	5,448	Yes	No

Supplementary Table 5: Genomes used in the genetic versus time and genetic versus geography correlation analysis of *Y. pestis*, *S. enterica* and *M. leprae* and their corresponding publication

Name	Species	Publication
BED030	*Yersinia pestis*	(67)
BED028	*Yersinia pestis*	((67)
BED034	*Yersinia pestis*	(67)
BED024	*Yersinia pestis*	(67)
BRA001	*Yersinia pestis*	(67)
LAI009	*Yersinia pestis*	(67)
LBG002	*Yersinia pestis*	(67)
MAN008	*Yersinia pestis*	(67)
NAB003	*Yersinia pestis*	(67)
NMS002	*Yersinia pestis*	(67)
STA001	*Yersinia pestis*	(67)
STN014	*Yersinia pestis*	(67)
STN020	*Yersinia pestis*	(67)
STN021	*Yersinia pestis*	(67)
STN019	*Yersinia pestis*	(67)
STN007	*Yersinia pestis*	(67)
STN002	*Yersinia pestis*	(67)
STN008	*Yersinia pestis*	(67)
STN013	*Yersinia pestis*	(67)
London_BD	*Yersinia pestis*	(65)
ELW098	*Yersinia pestis*	(68)
Barcelona	*Yersinia pestis*	(68)

OBS137	*Yersinia pestis*	(66)
OBS116	*Yersinia pestis*	(66)
OBS107	*Yersinia pestis*	(66)
OBS110	*Yersinia pestis*	(66)
OBS124	*Yersinia pestis*	(66)
MUR009	*Salmonella enterica*	(101)
MUR019	*Salmonella enterica*	(101)
IV3002	*Salmonella enterica*	(101)
OBP001	*Salmonella enterica*	(101)
IKI003	*Salmonella enterica*	(101)
SUA004	*Salmonella enterica*	(101)
MK3001	*Salmonella enterica*	(101)
ETR001	*Salmonella enterica*	(101)
Ragna	*Salmonella enterica*	(102)
Tepos_14	*Salmonella enterica*	(47)
Tepos_35	*Salmonella enterica*	(47)
3077	*Mycobacterium leprae*	(103)
Jorgen_625	*Mycobacterium leprae*	(103)
Refshale_16	*Mycobacterium leprae*	(103)
SK2	*Mycobacterium leprae*	(103)
SK8	*Mycobacterium leprae*	(103)
Body 188	*Mycobacterium leprae*	(104)
GC96	*Mycobacterium leprae*	(104)
Jorgen_404	*Mycobacterium leprae*	(104)

Jorgen_427	*Mycobacterium leprae*	(104)
Jorgen_507	*Mycobacterium leprae*	(104)
Jorgen_533	*Mycobacterium leprae*	(104)
Jorgen_722	*Mycobacterium leprae*	(104)
Jorgen_749	*Mycobacterium leprae*	(104)
SK11	*Mycobacterium leprae*	(104)
T18	*Mycobacterium leprae*	(104)
SK14	*Mycobacterium leprae*	(105)
SK27	*Mycobacterium leprae*	(105)

Datasets

Dataset 1: Summary statistics for the mapping to pCD1, pMT1 and pPCP1.
Dataset 2: Modern and ancient strains used in phylogenetic analysis.
Dataset 3: Yersinis pestis CO92 genes considered for the virulence analysis.
Dataset 4: Table containing all the genes present in the deletions detected in genomes in the LNBA lineage.
Dataset 5: SNP Effect analysis: Complete SNP Table with all SNPs and their effect.
Dataset 6: SNP Effect analysis: SNP Table with Intergenic SNPs unique to LNBA- lineage.
Dataset 7: SNP Effect analysis: SNP Table with Synonymous SNPs unique to LNBA- lineage.
Dataset 8: SNP Effect analysis: SNP Table with Non-synonymous SNPs unique to LNBA- lineage.
Dataset 9: SNP Effect analysis: SNP Table with Nonsense SNPs - Pseudogenisation events through STOP codon gain/loss or START Codon loss.
Dataset 10: SNP Effect analysis: Number of Pseudogenes per strain.
Dataset 11: Contaminant sites excluded from SNP analysis double-stranded libraries half-UDG with damage clipped (1bp at each end of the read clipped).
Dataset 12: Contaminant sites excluded from SNP analysis double-stranded libraries with damage.
Dataset 13: Contaminant site excluded from SNP analysis for samples with single-stranded libraries with damage.

SI References

1. B. Frohlich, *et al.*, “Bronze age burial mounds in the Khövsgöl aimag, Mongolia” in *Current Archaeological Research in Mongolia*, J. Bemmann, H. Parzinger, E. Pohl, D. Tseveendorzh, Eds. (Vor- und Frühgeschichtliche Archäologie Rheinische Friedrich-Wilhelms-Universität Bonn, 2009), pp. 99–116.
2. B. Frohlich, *et al.*, An overview of theories and hypothesis pertaining to Mongolian Bronze Age khirgisuurs in the Hovsgol Aimag, Mongolia. *Stud. Archaeol. INSTITUTI Archaeol. Acad. Sci. MONGOLICAE* **Tomus (IX) XXIX**, 123–143 (2010).
3. J. Littleton, *et al.*, Taphonomic analysis of Bronze Age burials in Mongolian khirigsuurs. *J. Archaeol. Sci.* **39**, 3361–3370 (2012).
4. C. Jeong, *et al.*, Bronze Age population dynamics and the rise of dairy pastoralism on the eastern Eurasian steppe. *Proc. Natl. Acad. Sci.* **115**, E11248 (2018).
5. P. Cholewa, A. Błażejewski, Sprawozdanie z badań wykopaliskowych stanowiska Chociwel 1, gm. Strzelin. *Śląskie Spraw. Archeol.* **36**, 107–115 (1995).
6. I. Lasak, Obiekty kultury unietyckiej z Chociwela, woj. wrocławskie. *Śląskie Spraw. Archeol.* **37**, 335–342 (1996).
7. P. Cholewa, L. Tymczyszyn, Sprawozdanie z ratowniczych prac wykopaliskowych na stanowisku 1 w Chociwlu w 1995 roku. *Śląskie Spraw. Archeol.* **38**, 117–126 (1996).
8. I. Lasak, Wyniki dalszych badań ratowniczych na cmentarzysku unietyckim Chociwel 1, gm. Strzelin. *Śląskie Spraw. Archeol.* **38**, 127–136 (1996).
9. K. Jarysz, R. Jarysz, P. Jarysz, Sprawozdanie z przeprowadzenia ratowniczych badań archeologicznych na obszarze objętym inwestycją pn. “Modernizacja systemu automatyki i budowa suszarni osadów na terenie oczyszczalni ścieków w Chociwelu, gm. Strzelin” (2012).
10. S. Rasmussen, *et al.*, Early Divergent Strains of Yersinia pestis in Eurasia 5,000 Years Ago. *Cell* **163**, 571–582 (2015).
11. D. Pokutta, *Population Dynamics, Diet and Migrations of the Únětice Culture in Poland* (2013).
12. M. E. Allentoft, *et al.*, Population genomics of Bronze Age Eurasia. *Nature* **522**, 167–172 (2015).
13. E. A. Kudryavtsev, *Archaeological Report: "Excavations of the archaeological heritage site “Grushevskoe settlement, VII-III centuries BC”* (2018).
14. Sabine. Reinhold, *Die Spätbronze- und frühe Eisenzeit im Kaukasus: materielle Kultur, Chronologie und überregionale Beziehungen* (R. Habelt, 2007).
15. E. Boulygina, *et al.*, Mitochondrial and Y-chromosome diversity of the prehistoric Koban culture of the North Caucasus. *J. Archaeol. Sci. Rep.* **31**, 102357 (2020).
16. L. Papac, *et al.*, Dynamic changes in genomic and social structures in third millennium BCE central Europe. *Sci. Adv.* **7**, eabi6941 (2021).
17. D. Daněček, J. Klementová, “Hostivice, k. ú. Hostivice, výstavba logistického centra Tulipán Park (př. č. 1/2007)” in *Archeologické Výzkumy Středočeského Muzea v Roztokách u Prahy v Roce 2007*, (Středočeský vlastivědný sborník, 2008), pp. 104–105.
18. J. Fernández-Eraso, *et al.*, Beginnings, settlement and consolidation of the production economy in the Basque region. *Quat. Int.* **364**, 162–171 (2015).
19. J. Fernández-Eraso, *et al.*, “Rediles y pastores en la Prehistoria Reciente de Rioja Alavesa” in *Miscelánea En Homenaje a Lydia Zapata Peña (1965-2015)*, (Servicio Editorial de la Universidad del País Vasco, 2017), pp. 393–426.
20. J. Rofes, *et al.*, Paleoenvironmental reconstruction of the early Neolithic to middle Bronze

Age Peña Larga rock shelter (Álava, Spain) from the small mammal record. *Quat. Res.* **79**, 158–167 (2013).
21. J. Fernández-Eraso, J. A. Mujika-Alustiza, La estación megalítica de la Rioja Alavesa: cronología, orígenes y ciclos de utilización. *Zephyrvs* **71**, 89–106 (2013).
22. M. Lipson, *et al.*, Parallel palaeogenomic transects reveal complex genetic history of early European farmers. *Nature* **551**, 368–372 (2017).
23. I. Olalde, *et al.*, The genomic history of the Iberian Peninsula over the past 8000 years. *Science* **363**, 1230–1234 (2019).
24. I. Mathieson, *et al.*, The Genomic History of Southeastern Europe. *Nature* **555**, 197–203 (2018).
25. D. Ya. Telegin, *Neolithic cemeteries of Mariupol type. [Neoliticheskie mogil'niki mariupol'skogo tipa]* (Naukova Dumka, 1991).
26. K. Massy, *Die Gräber der Frühbronzezeit im südlichen Bayern - Untersuchungen zu den Bestattungs- und Beigabensitten sowie gräberfeldimmanenten Strukturen. Mit Beiträgen von Nadja Hoke, Anja Staskiewicz, Wolf-Rüdiger Teegen und Stephanie Panzer.* (Materialhefte zur Bayerischen Archäologie, 2018).
27. A. Mittnik, *et al.*, Kinship-based social inequality in Bronze Age Europe. *Science* **366**, 731–734 (2019).
28. Ja. B. Berezin, A. A. Kalmykov, "Kurgan u sela Krasnogvardejskoe Stavropol'skogo kraja" in *Materialy Po Izučeniju Istoriko-Kul'turnogo Nasledija Severnogo Kavkaza*, (Archeologija 1, 1998), pp. 55–95.
29. A. Z. Beisenov, *et al.*, First Radiocarbon Chronology for the Early Iron Age Sites of Central Kazakhstan (Tasmola Culture and Korgantas Period). *Radiocarbon* **58**, 179–191 (2016).
30. А. З. Бейсенов, Тасмолинские Погребения Могильника Кызыл. 139–149 (2018).
31. G. A. Gnecchi-Ruscone, *et al.*, Ancient genomic time transect from the Central Asian Steppe unravels the history of the Scythians. *Sci. Adv.* **7**, eabe4414 (2021).
32. M. Ernée, *et al.*, *Mikulovice. Pohřebiště starší doby bronzové na Jantarové stezce - Mikulovice. Early Bronze Age Cemetery on the Amber Road* (Archeologický ústav Akademie věd České republiky, Praha, v.v.i., 2020).
33. S. Wirth, in *Das archäologische Jahr in Bayern 2006*, (Theiss, Konrad, 2007), pp. 53–56.
34. P. Limburský, "Pohřebiště kultury se zvoncovitými poháry ve Vlíněvsi. K problematice a chronologii konce eneolitu a počátku doby bronzové – The Bell Beaker cemetery in Vlíněves," Filozofická Fak., Univ. Karlova, Praha. (2012).
35. P. Limburský, *et al.*, *Pohřební areály únětické kultury ve Vliněvsi* (Archeologický ústav Akademie věd České republiky, Praha, v.v.i., 2018).
36. M. Dobeš, P. Limburský, Pohřebiště staršího eneolitu a šňůrové keramiky ve Vliněvsi – Gräberfeld des älteren Äneolithikums und der Schnurkeramik in Vliněves. Archeologické studijní materiály 22. Praha. *Archeol. Stud. Materiály* **22** (2013).
37. M. Dobeš, P. Stránská, R. Křivánek, P. Limburský, Časně eneolitické ohrazení ve Vliněvsi. Příspěvek k povaze kontaktu mezi jordanovskou a michelsberskou kulturou v Čechách – Frühäneolithisches Grabenwerk in Vliněves. *Památky Archeol.* **107**, 51–115 (2016).
38. M. Dobeš, P. Limburský, "Vliněves (Tschechische Republik) während Äneolithikum und Frühbronzezeit" in *Der Aufbruch Zu Neuen Horizonten. Neue Sichtweisen Über Die Europäischen Frühbronzezeit. Abschlusstagung Der Forschergruppe FOR550 Vom 26. Bis 29. November 2010 in Halle (Saale)*, Tagungen des Landesmuseums für Vorgeschichte Halle., H. Meller, F. Bertemes, Eds. (Landesamt für Denkmalpflege und Archäologie Sachsen-Anhalt Landesmuseum für Vorgeschichte, 2019), pp. 395–403.
39. G. U. Neumann, A. Andrades Valtueña, J. A. Fellows Yates, R. Stahl, G. Brandt, Tooth Sampling from the inner pulp chamber for ancient DNA Extraction (2020) https:/doi.org/10.17504/protocols.io.bqebmtan (March 24, 2021).
40. I. Velsko, E. Skourtanioti, G. Brandt, Ancient DNA Extraction from Skeletal Material (2020)

https:/doi.org/10.17504/protocols.io.baksicwe (October 30, 2020).
41. J. Dabney, *et al.*, Complete mitochondrial genome sequence of a Middle Pleistocene cave bear reconstructed from ultrashort DNA fragments. *Proc. Natl. Acad. Sci.* **110**, 15758–15763 (2013).
42. M. Meyer, M. Kircher, Illumina Sequencing Library Preparation for Highly Multiplexed Target Capture and Sequencing. *Cold Spring Harb. Protoc.* **2010**, pdb.prot5448 (2010).
43. N. Rohland, E. Harney, S. Mallick, S. Nordenfelt, D. Reich, Partial uracil-DNA-glycosylase treatment for screening of ancient DNA. *Philos. Trans. R. Soc. Lond. B. Biol. Sci.* **370**, 20130624 (2015).
44. F. Aron, G. U. Neumann, G. Brandt, Half-UDG treated double-stranded ancient DNA library preparation for Illumina sequencing (2020) https:/doi.org/10.17504/protocols.io.bmh6k39e (March 24, 2021).
45. M.-T. Gansauge, A. Aximu-Petri, S. Nagel, M. Meyer, Manual and automated preparation of single-stranded DNA libraries for the sequencing of DNA from ancient biological remains and other sources of highly degraded DNA. *Nat. Protoc.* **15**, 2279–2300 (2020).
46. R. Hübler, *et al.*, HOPS: automated detection and authentication of pathogen DNA in archaeological remains. *Genome Biol.* **20**, 280 (2019).
47. Å. J. Vågene, *et al.*, Salmonella enterica genomes from victims of a major sixteenth-century epidemic in Mexico. *Nat. Ecol. Evol.* **2**, 520 (2018).
48. D. H. Huson, *et al.*, MEGAN Community Edition - Interactive Exploration and Analysis of Large-Scale Microbiome Sequencing Data. *PLOS Comput. Biol.* **12**, e1004957 (2016).
49. A. Andrades Valtueña, *et al.*, The Stone Age Plague and Its Persistence in Eurasia. *Curr. Biol.* **27**, 3683-3691.e8 (2017).
50. J. A. Fellows Yates, *et al.*, Reproducible, portable, and efficient ancient genome reconstruction with nf-core/eager. *PeerJ* **9**, e10947 (2021).
51. M. Schubert, S. Lindgreen, L. Orlando, AdapterRemoval v2: rapid adapter trimming, identification, and read merging. *BMC Res. Notes* **9** (2016).
52. , Hannon, G.J. (2010) FASTX-Toolkit. http://hannonlab.cshl.edu/fastx_toolkit.
53. , BBMap. *SourceForge* (August 13, 2020).
54. S. Andrews, *et al.*, *FastQC* (2015).
55. H. Li, R. Durbin, Fast and accurate short read alignment with Burrows-Wheeler transform. *Bioinforma. Oxf. Engl.* **25**, 1754–1760 (2009).
56. H. Li, *et al.*, The Sequence Alignment/Map format and SAMtools. *Bioinformatics* **25**, 2078–2079 (2009).
57. , Picard Tools - By Broad Institute (March 12, 2020).
58. F. García-Alcalde, *et al.*, Qualimap: evaluating next-generation sequencing alignment data. *Bioinformatics* **28**, 2678–2679 (2012).
59. K. Okonechnikov, A. Conesa, F. García-Alcalde, Qualimap 2: advanced multi-sample quality control for high-throughput sequencing data. *Bioinformatics* **32**, 292–294 (2016).
60. J. Neukamm, A. Peltzer, *Integrative-Transcriptomics/DamageProfiler: DamageProfiler v0.4.9* (Zenodo, 2019) https:/doi.org/10.5281/zenodo.3557708 (August 14, 2020).
61. A. McKenna, *et al.*, The Genome Analysis Toolkit: A MapReduce framework for analyzing next-generation DNA sequencing data. *Genome Res.* **20**, 1297–1303 (2010).
62. K. I. Bos, *et al.*, Pre-Columbian mycobacterial genomes reveal seals as a source of New World human tuberculosis. *Nature* **514**, 494–497 (2014).
63. M. Keller, *et al.*, Ancient Yersinia pestis genomes from across Western Europe reveal early diversification during the First Pandemic (541–750). *Proc. Natl. Acad. Sci.* **116**, 12363–12372 (2019).
64. M. Feldman, *et al.*, A high-coverage Yersinia pestis Genome from a 6th-century Justinianic Plague Victim. *Mol. Biol. Evol.*, msw170 (2016).
65. K. I. Bos, *et al.*, A draft genome of Yersinia pestis from victims of the Black Death. *Nature*

478, 506–510 (2011).
66. K. I. Bos, *et al.*, Eighteenth century Yersinia pestis genomes reveal the long-term persistence of an historical plague focus. *eLife* **5**, e12994 (2016).
67. M. A. Spyrou, *et al.*, Phylogeography of the second plague pandemic revealed through analysis of historical Yersinia pestis genomes. *Nat. Commun.* **10**, 1–13 (2019).
68. M. A. Spyrou, *et al.*, Historical Y. pestis Genomes Reveal the European Black Death as the Source of Ancient and Modern Plague Pandemics. *Cell Host Microbe* **19**, 874–881 (2016).
69. N. Rascovan, *et al.*, Emergence and Spread of Basal Lineages of Yersinia pestis during the Neolithic Decline. *Cell* **176**, 295-305.e10 (2019).
70. J. Susat, *et al.*, A 5,000-year-old hunter-gatherer already plagued by Yersinia pestis. *Cell Rep.* **35**, 109278 (2021).
71. M. A. Spyrou, *et al.*, Analysis of 3800-year-old Yersinia pestis genomes suggests Bronze Age origin for bubonic plague. *Nat. Commun.* **9**, 2234 (2018).
72. H. Yu, *et al.*, Paleolithic to Bronze Age Siberians Reveal Connections with First Americans and across Eurasia. *Cell* **181**, 1232-1245.e20 (2020).
73. M. Eppinger, *et al.*, Draft Genome Sequences of Yersinia pestis Isolates from Natural Foci of Endemic Plague in China. *J. Bacteriol.* **191**, 7628–7629 (2009).
74. E. Garcia, *et al.*, Pestoides F, an Atypical Yersinia pestis Strain from the Former Soviet Union. *Genus Yersinia*, 17–22 (2007).
75. Y. Song, *et al.*, Complete Genome Sequence of Yersinia pestis Strain 91001, an Isolate Avirulent to Humans. *DNA Res.* **11**, 179–197 (2004).
76. Y. Cui, *et al.*, Historical variations in mutation rate in an epidemic pathogen, Yersinia pestis. *Proc. Natl. Acad. Sci.* **110**, 577–582 (2013).
77. W. Deng, *et al.*, Genome Sequence of Yersinia pestis KIM. *J. Bacteriol.* **184**, 4601–4611 (2002).
78. P. S. G. Chain, *et al.*, Insights into the evolution of Yersinia pestis through whole-genome comparison with Yersinia pseudotuberculosis. *Proc. Natl. Acad. Sci. U. S. A.* **101**, 13826–13831 (2004).
79. A. A. Kislichkina, *et al.*, Nineteen Whole-Genome Assemblies of Yersinia pestis subsp. microtus, Including Representatives of Biovars caucasica, talassica, hissarica, altaica, xilingolensis, and ulegeica. *Genome Announc.* **3** (2015).
80. A. A. Kislichkina, *et al.*, Nine Whole-Genome Assemblies of Yersinia pestis subsp. microtus bv. Altaica Strains Isolated from the Altai Mountain Natural Plague Focus (No. 36) in Russia. *Genome Announc.* **6** (2018).
81. A. A. Kislichkina, *et al.*, Eight Whole-Genome Assemblies of Yersinia pestis subsp. microtus bv. caucasica Isolated from the Common Vole (Microtus arvalis) Plague Focus in Dagestan, Russia. *Genome Announc* **5**, e00847-17 (2017).
82. E. Zhgenti, *et al.*, Genome Assemblies for 11 Yersinia pestis Strains Isolated in the Caucasus Region. *Genome Announc.* **3**, e01030-15 (2015).
83. G. A. Eroshenko, *et al.*, Yersinia pestis strains of ancient phylogenetic branch 0.ANT are widely spread in the high-mountain plague foci of Kyrgyzstan. *PLOS ONE* **12**, e0187230 (2017).
84. V. V. Kutyrev, *et al.*, Phylogeny and Classification of Yersinia pestis Through the Lens of Strains From the Plague Foci of Commonwealth of Independent States. *Front. Microbiol.* **9** (2018).
85. P. Cingolani, *et al.*, A program for annotating and predicting the effects of single nucleotide polymorphisms, SnpEff. *Fly (Austin)* **6**, 80–92 (2012).
86. J. T. Robinson, *et al.*, Integrative genomics viewer. *Nat. Biotechnol.* **29**, 24–26 (2011).
87. R. Bouckaert, *et al.*, BEAST 2.5: An advanced software platform for Bayesian evolutionary analysis. *PLOS Comput. Biol.* **15**, e1006650 (2019).
88. A. Rambaut, T. T. Lam, L. Max Carvalho, O. G. Pybus, Exploring the temporal structure of

heterochronous sequences using TempEst (formerly Path-O-Gen). *Virus Evol.* **2** (2016).
89. S. Kumar, G. Stecher, K. Tamura, MEGA7: Molecular Evolutionary Genetics Analysis Version 7.0 for Bigger Datasets. *Mol. Biol. Evol.* **33**, 1870–1874 (2016).
90. K. I. Bos, *et al.*, Paleomicrobiology: Diagnosis and Evolution of Ancient Pathogens. *Annu. Rev. Microbiol.* **73**, 639–666 (2019).
91. R. E. Kass, A. E. Raftery, Bayes Factors. *J. Am. Stat. Assoc.* **90**, 773–795 (1995).
92. A. R. Quinlan, I. M. Hall, BEDTools: a flexible suite of utilities for comparing genomic features. *Bioinformatics* **26**, 841–842 (2010).
93. R Development Core Team, *R: A Language and Environment for Statistical Computing* (R Foundation for Statistical Computing, 2008).
94. H. Wickham, *ggplot2: Elegant Graphics for Data Analysis* (Springer-Verlag New York, 2009).
95. B. Rudis, B. Bolker, J. Schulz, *ggalt: Extra Coordinate Systems, "Geoms", Statistical Transformations, Scales and Fonts for "ggplot2"* (2017). https://CRAN.R-project.org/package=ggalt.
96. E. Paradis, K. Schliep, ape 5.0: an environment for modern phylogenetics and evolutionary analyses in R. *Bioinformatics* **35**, 526–528 (2019).
97. R. J. Hijmans, *geosphere: Spherical Trigonometry* (2019).
98. J. Oksanen, *et al.*, *vegan: Community Ecology Package* (2020).
99. A. Kassambara, *ggpubr: "ggplot2" Based Publication Ready Plots* (2020).
100. J. Haiko, M. Kukkonen, J. J. Ravantti, B. Westerlund-Wikström, T. K. Korhonen, The Single Substitution I259T, Conserved in the Plasminogen Activator Pla of Pandemic Yersinia pestis Branches, Enhances Fibrinolytic Activity. *J. Bacteriol.* **191**, 4758–4766 (2009).
101. F. M. Key, *et al.*, Emergence of human-adapted Salmonella enterica is linked to the Neolithization process. *Nat. Ecol. Evol.* **4**, 324–333 (2020).
102. Z. Zhou, *et al.*, Pan-genome Analysis of Ancient and Modern Salmonella enterica Demonstrates Genomic Stability of the Invasive Para C Lineage for Millennia. *Curr. Biol.* **28**, 2420-2428.e10 (2018).
103. V. J. Schuenemann, *et al.*, Genome-Wide Comparison of Medieval and Modern Mycobacterium leprae. *Science* **341**, 179–183 (2013).
104. V. J. Schuenemann, *et al.*, Ancient genomes reveal a high diversity of Mycobacterium leprae in medieval Europe. *PLOS Pathog.* **14**, e1006997 (2018).
105. T. A. Mendum, *et al.*, Mycobacterium leprae genomes from a British medieval leprosy hospital: towards understanding an ancient epidemic. *BMC Genomics* **15**, 270 (2014).
